# Citizens at the forefront of the constitutional debate: Voluntary citizen participation determinants and emergent content in Chile

**DOI:** 10.1371/journal.pone.0267443

**Published:** 2022-06-06

**Authors:** María Paz Raveau, Juan Pablo Couyoumdjian, Claudio Fuentes-Bravo, Carlos Rodriguez-Sickert, Cristian Candia

**Affiliations:** 1 Centro de Investigación en Complejidad Social (CICS), Facultad de Gobierno, Universidad del Desarrollo, Santiago, Chile; 2 Centro de Políticas Públicas, Facultad de Gobierno, Universidad del Desarrollo, Las Condes, Chile; 3 Facultad de Economía y Negocios, Universidad del Desarrollo, Las Condes, Chile; 4 Facultad de Derecho, Universidad de Chile, Santiago, Chile; 5 Data Science Institute, Facultad de Ingeniería, Universidad del Desarrollo, Las Condes, Chile; 6 Northwestern Institute on Complex Systems (NICO), Northwestern University, Evanston, IL, United States of America; University of Catania, ITALY

## Abstract

In the past few decades, constitution-making processes have shifted from being undertakings performed by elites and closed off from the public to ones incorporating democratic mechanisms. Little is known, however, about the determinants of voluntary public participation and how they affect the outcomes of the deliberative process in terms of content and quality. Here, we study the process of constituent involvement in the rewriting of Chile’s constitution in 2016. A total of 106, 412 citizens in 8, 113 different local encounters voluntarily congregated in groups of ten or more to collectively determine what social rights should be considered for inclusion in the new constitution, deliberating and then articulating in the written word why should be included. We brought our data to statistical regression models at the municipality level, the results show that the main determinants associated with increasing citizen participation are educational level, engagement in politics, support for the government, and Internet access. In contrast, population density and the share of Evangelical Christians in the general population decrease citizen participation. Then, we further analyze the written arguments for each collectively-selected constitutional rights. The findings suggest that groups from socioeconomically developed municipalities (with higher educational levels and where the main economic activities are more distant from natural resources), on average, deliberate consistently more about themes, concepts, and ideas compared to groups from less developed municipalities. These results provide an empirical ground on the driver factors of voluntary citizen participation and on the benefits and disadvantages of deliberative democracy. Hence, results can inform the organization of new deliberative processes.

## Introduction

The increasing rate of sociopolitical crises around the globe encourages us to rethink the mechanisms of democracy and how to improve its representativeness –how good the diversity is represented– and functionality –the quality of outcomes. The long tradition of constitutions produced by elite social classes is being challenged by a democratic constitution-making process because the former lacks legitimacy due to it consistently serving the needs of elites at the expense of the general population [1–3]. Deliberative democracy is emerging as an approach that fosters public involvement [4] where citizens can participate before, during and after the process of constructing the constitution that will govern their societies [2, 3, 5]. Yet, we still lack a full understanding of public participation in democratic and deliberative constitutional-making processes [4].

Here, we use data on Chile’s deliberative constitution-making process in 2016 to understand public participation in a deliberative democracy setting. In October 2015, the Chilean government proposed a new constitution-making process with the final aim of generating a new governing document. The Chilean constituent process of deliberation represents a deliberative democratic exercise in which citizens are active participants at the forefront of the debate that generates inputs for the proposed new constitution.

**Fig 1:**
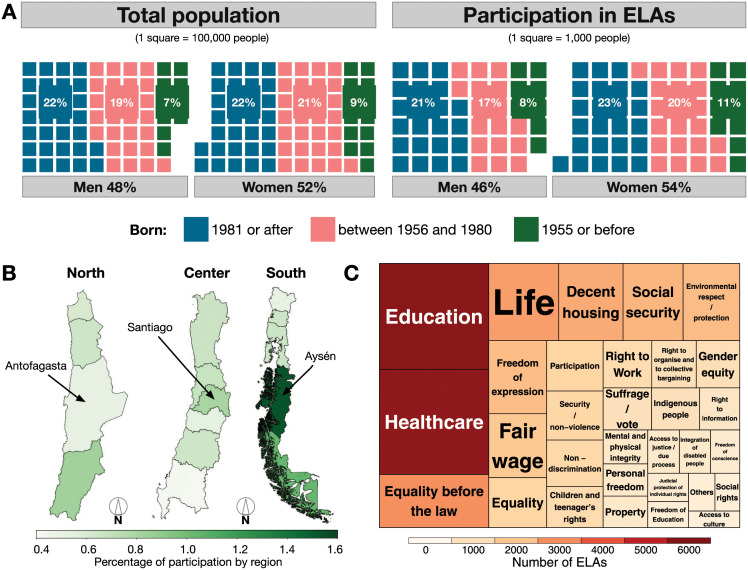
Citizen participation in ELAs. A) Composition of national population from Census 2017 data and of citizen participation in self-convoked encounters (ELAs), by gender and generational cohorts (colors). Participation distribution differs significantly from the population distribution. However, the difference is negligible according to the Cohen’s d standard. Participation distribution differ significantly from the population distribution. However, according to the Cohen’s d standard the difference is small. The contingency tables and chi-squared test results can be found in the appendix (S1 and S2 Tables in S1 File). B) Percentage of the population participating in ELAs per region in Chile. From left to right, the northern macro-zone, the center macro-zone, and the southern macro-zone. The map files are publicly available on the Library of the National Congress of Chile website. C) What constitutional rights do people select at the country level? The tree-map depicts the most selected constitutional rights in all the self-convoked encounters, ELAs. Color and size represent the number of ELAs in which those concepts were selected.

According to the OECD report [6], Chile’s case was unprecedented because of its high rate of participation (1.13%) across 98% of its territory (Fig 1B), ultimately involving 204, 402 individuals. Similar experiences in the constituent process in other countries resulted in lower rates of citizen participation. For instance, Colombia in 1991 achieved (0.06% citizen participation); Iceland in 2010, (0.3% citizen participation); and Tunisia in 2011, (0.06% citizen participation [6]). In terms of gender and age, the participation distribution in the Chilean deliberative process differed little from the population distribution, suggesting that it represents citizens at the country-level for these two dimensions (Fig 1A). However, even when it is small, this difference is statistically significant according to Cohen’s d standard.

The process started with government-convened sessions of civic education, which were not a prerequisite for the next steps. It was followed by a participatory phase divided into four stages: (i) an individual online questionnaire; (ii) local, deliberative self-convoked encounters (in Spanish *Encuentros Locales Autoconvocados*, henceforward “ELAs”); (iii) dialogues at the province level; and finally (iv) dialogues at the regional level. The three group-level instances (local, provincial, regional) were nested: the results of the local encounters would serve as the basis for the provincial process, and these, in turn, for the regional process, which the executive power coordinated last two.

The participants mostly organized ELAs. At each stage, citizens were asked to debate four constitutional dimensions: (i) Principles and Values, (ii) Rights, (iii) Duties, and (iv) Institutions. For each of these four constitutional dimensions, participants collectively selected seven constitutional concepts from a list provided by the government or determined new constitutional concepts to be considered after group deliberation (Fig 1C). The provided list of constitutional concepts was based on a comparative analysis of 16 constitutions (Argentina, Brazil, Canada, Chile, Colombia, Finland, France, Germany, Italy, Mexico, Netherlands, Spain, Sweden, Switzerland, United States of America, and Uruguay) [6]. Given the current increasing demands on social rights in Chile [7], we further focused on the constitutional dimension of *Rights* in all local self-convoked encounters. A total of 106, 412 citizens participated in 8, 113 ELAs [8], and each ELA consisted of between 10 to 30 people, all over 14 years old [9].

Participants wrote down a short argument explaining why this concept should be included in the new constitution for each chosen or determined concept. The set of prioritized concepts in each dimension, the arguments, and the ELA’s minute were finally uploaded to a platform created for this purpose. The result of this consultation served as an input to influence the process of the executive power writing the new constitution [6]. The proposal was submitted to Congress in March 2018 without any further advance. The social outbreak experienced in Chile in 2019 gave way to a new constituent process taking place in 2021 and 2022.

The whole process was organized and coordinated by the executive power. To oversee and guarantee the transparency of the consultation process and the lack of political biases, the President created a Citizen Council of Observers. This group consisted of 15 members from different backgrounds and political positions. There was also a Systematization Committee, an autonomous body in charge of processing the consultation results. The committee was composed of scholars from the Universidad de Chile, Pontificia Universidad Católica de Chile, and the United Nations Development Programme (UNDP).

Here, we study the determinants of citizen participation in ELAs by setting up various regression models at the municipality level. We note that Chile has 346 communes and 345 municipalities. In this work, we have exclude the data from the Chilean Antarctic Territory. Henceforward, we use the term municipality for simplicity. We included sociodemographic and political variables as well as social capital indicators from different data sources such as the census, the Electoral Service, and the National Municipal Information System. Regarding the influence of sociodemographic variables, one important finding is that engagement in politics and support for the government increase participation, suggesting that citizen involvement may be ideologically driven. Next, we analyzed the effects of the citizen participation determinants and other relevant variables on the content and quality of the arguments. To do so, we first identified the latent topics in the argument texts using structural topic modeling. Then, we explored the content changes for each topic for cities with different characteristics, showing that the emergent content can be ideologically differentiated. Finally, we proposed a metric to establish the quality of arguments based on lexical and syntactic markers and similar but non-repeated terms. Our results show that municipalities with higher educational levels and where the main economic activities are relatively distant from natural resources (both proxies for a municipality’s knowledge) deliberate on themes, concepts, and ideas, whereas municipalities with low education levels and where the main economic activities belong to the primary sector (extraction) deliberate more on actions without defending their points of view with themes, concepts, or ideas.

This paper is organized as follows. In the Introduction we review the main determinants of political participation, and the pros and cons of citizen participation in Constitution-making processes. The Methods section describes the statistical model used to evaluate the participation in ELAs and the content and quality of the arguments. Then we introduce our results on citizen participation, topic modeling and text analysis. Finally, we present our conclusions and findings.

### Political participation

In the last few decades, research on political participation has been mainly focused on voter turnout, engagement in political parties, and civil disobedience, categories in which the most important participation determinants are gender [10–12], age [13–15], education, income [12, 16, 17], and social capital [18–25].

Here, we inspect some of the main findings on political participation in our context of deliberative processes. We acknowledge that both forms of citizen participation—voting and public deliberation—will be similar in some aspects and different in others.

#### Gender

In Latin America, the World Economic Forum’s gender gap index shows low performance in political empowerment when correlated to economic participation and opportunity, educational attainment, and health [10]. Overall, Latin American women are less likely to be involved in political activities, particularly protest demonstrations and engagement with a political party. This phenomenon is closely related to women’s presences in the work force: in general, the greater the female labor force participation, the greater their political participation [10]. [11] studied gender, employment, and political participation found a number of work-related factors that enhance political activity, such as getting requests for political activity on the job, supervising others, and exercising civic skills. Negative work-related factors are also impactful: the experience of being discriminated against on the basis of sex can also lead to political activity. Additionally, marriage and having children at home indirectly depresses political activity because caring for young children often prevents a woman’s participation in the work force and political activity [11]. However, a recent study on electoral turnout in Chile shows that for almost all age groups, voter turnout rates for women exceed voter turnout rates for men [12].

#### Age

The relationship between voter turnout and age is well-established in the literature. The level of participation is relatively low during early adult life, gradually increases in middle age, and slowly declines with old age [13]. This behavior has been connected to a variety of stages in adult life such as marriage, home ownership, steady employment, and leaving school [14]. However, in Chile there is also a generational effect in voter turnout due to the electoral system. Those who registered to vote in the 1988 plebiscite were enrolled in a system with mandatory voting until 2011; the increase in electoral participation with age is therefore not directly associated with the age of voters but with their registration rates. (See subsection *Determinants of citizen participation* for more political context.) Those who reached the voting age after the plebiscite show lower enrollment and participation rates [15].

#### Education and income

Regarding socioeconomic resources, income and education level have a positive and significant effect on political participation. Economic and cultural resources engender the development of intellectual and cognitive skills that typically reduce the subjective costs of participation [16]. In Chile, the young electorate is strongly class-biased, particularly by income, which causes voter registration rates in the upper class to be double those in the lower class [17]. An analysis of the 2016 municipal elections in Chile shows a negative effect of income on voter turnout [12]. However, this effect reverses when the observations are limited to the Santiago Metropolitan Region. This implies that spacial socioeconomic segregation is stronger in Santiago than in the rest of Chile, where different socioeconomic classes coexist in the same municipality [12].

#### Social capital

The term social capital was introduced by [18] as a conceptual tool to describe how rational or purposive action is shaped by the social context. While physical capital improves tools to facilitate production and human capital promotes the development of new skills and capabilities, social capital relies on the relations between persons to facilitate increased engagement in a broad range of traditional political activities [19]. Empirical evidence supports this finding.

For instance, a study conducted using data from Argentina, Chile, Mexico, and Peru shows that greater involvement in non-political organizations and higher levels of interpersonal trust lead to more participation in political activities [20]. Population density and rurality have been used as proxies for social capital. Population density has a negative and significant effect on voter turnout [21, 22] while rurality has a positive but not significant effect on voter turnout [21, 22]. In other studies, exploration of the role of urban and rural residency in promoting political activity has reached mixed conclusions. While some studies have found that urbanization makes political participation easier [23, 24], others have shown that isolation and the lesser availability of public services in rural areas increase a sense of responsibility to others [25], which promotes social capital and, therefore, civic engagement.

The evidence in Chile shows a significant negative effect of population density on electoral participation but no significant effect from the rurality variable [21]. On the other hand, participation in voluntary associations is commonly used to measure social capital [26]. This idea is based on the assumption that membership in voluntary associations generates trust and facilitates cooperation among members. Also, citizens that join voluntary organizations usually meet more people and expand their social circle, and hence become more engaged in civic life [16]. However, participation in associations represents only a small part of human interaction. Participation in other networks such as family, schools, work, media and internet has a strong impact on norms and values [27]. Therefore, the role of associations in social capital generation may be limited. Recent research has addressed the role of media in the production of social capital. The evidence suggests that use of the Internet supplements network capital by extending existing levels of personal and telephone contact, thereby increasing social contact and civic engagement [28, 29]. Thus, the more people are involved in online organizational and political activities, the more they are involved in such activities offline [30].

### Citizen participation in constitution-making processes

Nowadays, it is widely believed that democratic constitutions should be created and adopted through democratic processes because elite-made constitutions suffer from a lack of legitimacy by representing the interests of the elites, often to the detriment to the interests of the people they govern [1–3]. This idea relies on the belief that the sense of ownership that comes from sharing authorship enhances the understanding, respect for, and support of the constitution’s constraints. These assumptions, however, have only recently been significantly tested by any rigorous study of the precise relationships between constituents’ participation and a constitution-making processes [2], as have this idea’s effects on democratic governance. A twelve-country study, commissioned by the Institute for Democracy and Electoral Assistance (IDEA), found that “more representative and inclusive constitution building processes resulted in constitutions favoring free and fair elections, greater political equality, more social justice provisions, human rights protections, and stronger accountability mechanisms” [31]. Later, researchers found that the use of public referenda during the process makes constitutions more likely to include every category of right and to provide for universal suffrage, a secret ballot, a referendum process in ordinary government, and a public role in approving constitutional amendments [2].

This new constitution-making process that incorporates citizen participation before, during, and after the text is finalized is supported by numerous international organizations and entities, including the European Union, the Commonwealth Human Rights Initiative, the United States Institute of Peace, and the Centre for Democracy and Development [5]. Models of participation differ considerably, not only among countries, but within a country. The modal form of participation in constitutional design is the approval by referendum of the final document as a whole [2]. Other forms of participation include elections for constitution-making assemblies; civic education and media campaigns using newspapers, radio, television, web sites, and public meetings; prior agreement on broad principles as a first phase of constitution making; an interim constitution to create space for longer term democratic deliberation; and public deliberation [2, 3].

Public deliberation is particularly controversial. Theoretically, group discussions are more likely to produce better outcomes. The idea of sharing different perspectives to formulate political decisions has interested numerous thinkers, including Aristotle, who wrote: “The people, when they are assembled, have a combination of qualities which enables them to deliberate wisely and to judge soundly” (*Pol*.III.11.1281 a12-b10 [32]). Nowadays, deliberative democracy is a field of study by itself. According to Habermas [33], given the proper conditions of an inclusive and critical discussion in an argumentative form free of any internal or external coercion, public deliberation should reach consensus. In this context the idea of consensus is key, for it confers legitimacy to any political result and, in this case, to a constitution. To Bernard Manin, the essential condition for legitimacy is public deliberation itself. Thus, a decision is legitimate not because it represents the will of all, but because it results from the deliberation of all [34]. A more moderate view (which reconciles pluralism and consensus) accepts a meta-consensus instead of a consensus on the outcome. Meta-consensus can refer, for example, to agreement on the nature of the issues to discuss or on the domain of relevant reasons or consideration to be considered in the discussion [35]. In any case, whether consensus is reached or not, public deliberations have the potential to improve individual rationality [36], a positive effect. Furthermore, deliberation has an emancipatory effect [37], by which the participants develop integrative thinking on the discussed issues, overcoming preexisting distortions.

However, for deliberation to be successful, no information asymmetry can exist. As pointed out by Weinshall [32, 38], “To claim that an instrumentally rational will be produced by democratic deliberation or discourse requires one to assume that the public is either very well informed or that it is capable of becoming adequately informed.” If these assumptions fail, allowing public participation will not make rational outcomes more likely [38]. Other criticism focuses on the “group polarization effect,” by which the members of a deliberating group push their initial individual positions to extremes though the process of deliberation [39, 40], as well as on the instability and inconsistency of citizens’ political preferences [41], and the effect of cognitive biases [42]. Another concern has to do with the size of deliberating groups and the difficulty of reaching agreement. In larger groups, the costs of negotiation increase, making the *status quo* more difficult to change. This is particularly impactful when participants or groups have veto powers over the adoption of new rules [43].

We have reviewed several pros and cons of public deliberation, but in our inquiry into public deliberation, we focus on the Public Ignorance Objection [44, 45]. According to Somin (1998), voters are ignorant about specific policies and the basic structure and functioning of government. Furthermore, most voters do not have a single analytical framework—a few basic principles—to integrate different political issues [45]. In the same vein, [44] identifies two types of ignorance. The first, Belief Ignorance, consists of the possession of false beliefs to such a degree that one cannot reach a correct conclusion. If the beliefs or premises are true, however, the conclusions will also be true, and so the presence of this type of ignorance alone is not enough to reject deliberative democracy. The second, Agent Ignorance, is a scenario in which the citizen, despite having the correct information on a given issue, reaches the wrong conclusions. This type of ignorance, “would prove devastating to any conception of democracy, not just deliverativist versions” [44].

How can we quantify the extent of public ignorance in ELAs? In practice, none of the other types of ignorance can be tested; that would require evaluating the veracity of each argument text, and since most of them are deontic sentences–an expression of practical reasoning that cannot be verified because it expresses a desire whose content is a future action–this cannot be done. We can, however, consider the quality of the argument. A truly rational deliberation needs individuals to communicate high-quality arguments that are suitable for evaluation and counterargument. But there can be a relevant number of low-quality arguments—that is, arguments that cannot be contrasted and that do not contribute to the objective of group deliberation and thus to the epistemic improvement of individual reasoning [36]. In this case, the result of the deliberation will probably not be as good as the deliberative democracy theorists expect. Although low-quality arguments do not necessarily imply false beliefs, they often lead to faulty or sub-optimal deliberations. In this work, we evaluate the quality of ELAs’ argument texts by looking at justifications or testimonies in those deliberations. In this sense, we are not linking the quality of arguments to the possibility of verifying their contents. Instead, we will look for a functional quality related to the capacity of a sentence to contribute to the deliberation with useful information. In any case, low-quality arguments still have a value: they inform about the agent’s ideological position on a particular issue.

## Methods

The new constitution-making process held in Chile between April and August 2016 is particularly important worldwide because of its high levels of citizen participation and geographic coverage. In the entire four-stages of the process, more than 200, 000 citizens participated. Here, we focus on the ELA stage. For each ELA, the data includes the name of the commune in which the encounter took place, the age and gender of participants, the collectively agreed-upon constitutional rights, and the argument texts. We ran several statistical models to explain the number of ELAs held in each municipality. The data on ELAs used in this study is publicly available and was previously systematized, and all the argument texts were normalized [46, 47]. Finally, we used complementary data on the 345 Chilean municipalities.

During the ELA meetings, the participants were asked to identify the most important constitutional concepts in four dimensions: Rights, Values and Principles, Duties, and Institutions. Given the recent social movement in Chile and the increasing demand for social rights [7], we focused our analysis on the constitutional concepts within the Rights dimension. The original list of rights provided by the government included 45 items, and ELA participants added 14 new rights in the process, resulting in 59 rights in total. Among the new concepts, Social Rights, Standard of Living, and the Right to Quality Public Health Care reaffirm the increasing demands for social rights in Chile, while Respect Life from Conception and the Right to Make One’s Own Decisions About One’s Life arise as specifications of the original Right to Life. Fig 1C shows that most rights deliberated upon and accepted in the ELAs are rights that would exist at the national level. The full list of rights available to ELA participants, along with the new concepts that emerged in the deliberation process, can be found in the appendix. For each selected constitutional right, each ELA wrote down a short argument about why that concept is relevant and why it should be included in the new constitution. These texts were subsequently processed and classified by combining experts’ knowledge and machines tools by [46, 47].

Even when these texts may not literally follow the canonical argument structure—i.e., one or more premises followed by one or more conclusions-we treated them as arguments. The reason behind this is that the argument structure can be reconstructed by the context. Thus, the participants expressed their point of view on public matters, and the texts they wrote can be understood as the premise to the conclusion of selecting a concept to be included in the new constitution [48, 49].

### Determinants of self-convoked citizen participation

#### Statistical model

According to [50], historical events are associated with recurring generational archetypes. Therefore, we define age cohorts based on the Chilean political background. In 1973 the Chilean military, led by general Augusto Pinochet, staged a *coup d’état* against the socialist government of Salvador Allende. An authoritarian military regime ruled the country until the transition to democracy in 1989. It has been found that people in younger cohorts are more likely to be less politicized than people in older ones, since they did not experience the military dictatorship nor the political climax that prevailed during the transition to democracy [51]. Here we use the three age cohorts proposed by [52]: (i) Chileans who were over 18 years old when Pinochet took control in 1973; (ii) those born between 1956 and 1980 and who experienced life under the dictatorship until Pinochet’s ousting in 1998; and (iii) those born in 1981 and after who became adults after the reestablishment of democracy.

Fig 1A shows the composition of the national population and citizen participation in ELAs by gender and generational cohorts. A chi-squared test was performed to test whether the distributions of age and gender are statistically equivalent between the national population and citizen participation in ELAs. In either case, we can reject the test’s null hypothesis; therefore, the participation distribution differs from the population distribution at the 1% significance level. However, according to the Cohen’s d standard, the difference is small. The contingency tables and chi-squared tests results can be found in the appendix (S1 and S2 Tables in S1 File). In contrast, the share of population participating in ELAs is substantively different when measured according to geography. Fig 1B shows citizen participation at the regional level, and the same variability is observed at the municipality level.

Using ordinary least squares (OLS) models, we studied the effects of sociodemographic and political variables on citizen participation. Given that each ELA represents an instance of collective deliberation, we measured citizen participation as the total number of ELAs held in each municipality instead of the total number of participants in each municipality. Nevertheless, the main findings of this work do not significantly change when using the number of participants as a dependent variable (see S8 Table in S1 File). A histogram of the ELAs’ number of participants at the national level is also provided in S1 Fig of S1 File. The regression model specification is the following:

$$\begin{array}{c} \text{log}\left(1+ELA_{i}\right)=\beta X_{i}+e_{i}, \end{array}$$ (1)

where *ELA*_*i*_ is the total number of ELAs held in the municipality *i*, *X*_*i*_ is a vector of covariates, and *e*_*i*_ is a random error term. The coefficients of interest are the *β*s, which measure the effect of the covariates on citizen participation. Note that we add 1 unit to the dependent variable. This correction serves to account for zeroes at the logarithmic transformation [53].

#### Independent variables

We did not seek any any personal information about income, education, or political inclination from participants. Instead, we run municipality-level models and aggregated data on socioeconomic and demographic indicators such as population, rurality, population density, education, socio-demographic classification, and Internet penetration rate, among others. When analyzing the collective selection of constitutional rights we use a socioeconomic development index (SEDI) that is proposed by the Public Health Observatory in Chile and comprised of education, income, poverty and housing materiality [54]. For social capital, we used the number of community organizations in the municipality, such as parent centers, cultural centers, sport clubs, and others. We also included political variables such as voter turnout, party affiliation, and political orientation to assess the impact of political engagement. Finally, we included variables relative to the municipality in order to test whether people were mobilized to participate. Because evidence suggests that participation in Evangelical Christianity can provide skills that members can transfer to political activity [55], we added an additional variable to account for the share of Evangelical Christians living in the municipality.

We collected all the independent variables from official national sources like the population and housing census (CENSO), the Electoral Service (SERVEL), the National Municipal Information System (SINIM), the National Office for Regional Development (SUBDERE), and the National Socioeconomic Characterization Survey (CASEN). Table 1 presents a detailed description of those variables. The descriptive statistics and the correlation for the most relevant variables can be found in the appendix (S4 Table and S2 Fig in S1 File, respectively).

**Table 1:** Variable description and data.

Name	Description	Source	Year	N	Type
Population	Municipal population over 14 years old (only people over the age of 14 were allowed to participate in an ELA).	CENSO	2017	345	Count
Higher education	Share of municipal population who have successfully completed a higher education degree (advanced technician, bachelor, MSc, PhD.)	CENSO	2017	345	Continuous (0–1)
Internet penetration rate	Constructed as the interaction of two variables from CASEN survey: (i) share of households with at least one internet-connected device, (ii) number of different uses of Internet.	CASEN	2015	324	Continuous (0–1)
SUBDERE groups	Socio-demographic municipal classification based on the dependence on the municipal common fund and the local population. This typology divides municipalities in 7 groups plus an exception group with the highest income municipalities. Group 1 is the most vulnerable, i.e., municipalities in this group have less population and show higher dependency on the municipal common fund.	SUBDERE	2005	335	Categorical
Poverty	Income poverty rate by municipality, based on income information from CASEN survey, using a method of Small Area Estimation (SAE).	INE	2017	344	Continuous (0–1)
SEDI	Socio-Economic Development Index, which comprises education, income, poverty, housing and sanitation.	OCHISAP	2013	324	Continuous (0–1)
Community organizations	Number of community organizations in the municipality, such as parent centers, cultural centers, sport clubs, among others.	SINIM	2015	342	Count
Participation in comm. org.	Share of municipal population that declares to participate in a community organization	CASEN	2015	324	Continuous (0–1)
Population density	Number of people living in the municipality, per square kilometer (km^2^)	SINIM	2015	345	Continuous
Rurality	Share of municipal population living in rural areas. A rural area is defined as an agglomeration with more than 1,000 inhabitants, or between 1,001 and 2,000 inhabitants where more than 50% of the economically active population is engaged in primary economic activities.	CENSO	2017	345	Continuous (0–1)
Born in 1981 or after	Share of municipal population born in 1980 or after, which represents the youngest cohort of our study.	CENSO	2017	345	Continuous (0–1)
Women	Proportion of women in the municipality	CENSO	2017	345	Continuous (0–1)
Single-parent family with children	Share of single-parent families, with children, in the municipality.	CENSO	2017	345	Continuous (0–1)
Two-parent family with children	Share of two-parent families, with children, in the municipality.	CENSO	2017	345	Continuous (0–1)
Party affiliation	Share of municipal population affiliated to any political party.	SERVEL	2016	345	Continuous (0–1)
Voter turnout	Voter turnout in 2013 presidential elections at the municipal level.	SERVEL	2013	345	Continuous (0–1)
Votes for standing president	Share of votes received by the winning candidate in 2013 presidential elections by municipality.	SERVEL	2013	345	Continuous (0–1)
Municipal officials	Share of municipal population employed by the city hall.	SINIM	2015	345	Continuous (0–1)
Mayor	3 dummy variables to take into account the political party which supported the winning candidate for mayor, during the 2012 municipal elections: the first one is equal to 1 if the party is within the government coalition, and 0 otherwise; the second one is equal to one if the party is in the opposition, and 0 otherwise; the third dummy variable is assigned to 1 when the mayor ran for office with no formal party support.	SERVEL	2012	345	Categorical
Incumbent mayor	Dummy variable that takes the value 1 if an incumbent mayor is reelected, and 0 otherwise.	SERVEL	2012	345	Categorical
Government influence	Consists of the sum of: (i) the share of votes obtained by the pro-government deputies, relative to the total votes obtained by both elected deputies; (ii) 1, if the mayor was supported by the government coalition, and 0 otherwise.	SERVEL	2012	345	Continuous (0–2)
Evangelical Christians	Share of municipal population who declared to profess an evangelical Christian religion.	CENSO	2012	341	Continuous (0–1)

Notes: (i) The administrative division of Chile consists of 346 municipalities, from which we excluded the municipality of Antártica because of its special situation. (ii) Sources: Population and housing census (CENSO); Electoral Service (SERVEL); National municipal information system (SINIM); National office for regional development (SUBDERE); National Socio-Economic Characterization Survey (CASEN); Public Health Observatory in Chile (OCHISAP); National Institute of Statistics (INE). (iii) CASEN survey lacks representativity at municipal level; (iv) Small-area estimation (SAE) refers to methods to address the limitations of survey data to produce reliable estimates of poverty for different geographical locations. The methodology used in Chile combines data from CASEN survey and Census.

### Latent topics of deliberative arguments

The analysis of large text corpora has proven fruitful in studying a range of topics in social sciences, including the analysis of discourse surrounding social movements [56], content analysis for political texts [57, 58], mapping social conflicts from legal sentences [59, 60], research choice and the production of scientific knowledge [61], and communication and collaboration within organizations [62] (For a review of the history of content and text analysis in sociology and the social sciences, see [63].)

Topic modeling, a group of inductive techniques used to discover hidden topics contained in text documents, has been particularly effective in analysis of this corpora. In this frame, a corpus is a collection of *M* documents (*d*) denoted by *D* = *d*_1_, *d*_2_, …*d*_*M*_, while a document is a sequence of *N* words denoted by *w* = (*w*_1_, *w*_2_…*w*_*n*_), where *w*_*n*_ is the *n*-th word in the sequence. In general, models within this category assume that each document in a text corpus is produced from a mixture of latent topics, which in turn consists of a collection of words. Topics are defined as the set of elements that can represent a theme present in a collection of documents without loss of statistical information [64]. In our particular context, a document corresponds to an argument text.

Among topic models, Structural Topic Modeling (STM) is a technique enabling researchers to incorporate document-level metadata information into analysis. The goal of STM is to discover topics and estimate their relationship to document metadata, conducting hypothesis testing of these relationships. STM has been used to find latent topics in social scientific research [65, 66], analyze open-ended survey responses [67], and to uncover the underlying network structures of groups and communities by identifying topics in publications regularly consulted by those groups [68].

To implement STM analysis, we use the standard *stm* R package developed by [69], and we follow a conventional approach. First, stop-words are filtered from texts [70], then texts are tokenized and all the punctuation is removed [71]. Finally, bi-grams and tri-gramas are created [72] to capture significant co-occurrences of words. After pre-processing texts, we run the STM model where texts’ metadata can be incorporated in two ways: (i) as topical prevalence, which refers to how much of a document is associated with a topic, and (ii) as topical content, which is related to the words used within a topic. The generative process incorporates the *p* covariates in a 1-by-*p* vector of document covariates *X*_*d*_ for document *d*.

### Text quality of deliberative arguments

Finally, to assess the quality of argument texts we propose a metric with two categories: (i) texts that offer only an assertion without adding a justification, and (ii) texts that offer an assertion and also justify the content with testimony or evidence of some kind. From these two categories, the former will be considered as low-quality argument. For example, “We must preserve the environment for future generations” would be a low-quality argument, and “It is very important to live in a pollution-free environment and for that we must promote respect for nature, both as citizens and businessmen” would not be. In the second case there is a justification to promote respect for nature, which is the importance of living in a pollution-free environment. To identify the presence of evidence or testimony, we use all available lexical and syntactic markers (argumentative, causal and conclusive connectors; evidential markers; etc.) that allow us to isolate those sentence components. As we cannot rely on these markers nor on punctuation, we also consider the presence of two or more (non-repeated) related terms as an indicator of a subordinate clause. We note that restricting this criteria to three related terms does not alter the significance (or non-significance) of our results. If we perform this analysis by topic, we can extract these terms from the list of topic words. For each topic, we built a list with the first 30 highest probability n-grams plus the 30 first frequent and exclusive n-grams. We then deleted non-informative verbs and adjectives/adverbs (such as “must” or “same”), and also n-grams when all of their components were already included in the list as tokens.

The quality of argument texts is also complemented with the degree of conceptualization of the topic. Words have different psychological properties and often are processed in the brain very differently [73, 74]. Typically, a content/style classification is used to distinguish between *what* people are saying (i.e., the content of a communication), and *how* people are communicating (i.e., the style words such as prepositions, conjunctions, articles, and auxiliary verbs). Since most style words—including nouns, adjectives, and adverbs—have been removed from the corpus for the purpose of the topic modeling, here we classify them according to function: action words and phrases and concept words and phrases. For example, all terms containing verbs like “must,” “be able to live,” or “there must be education” are classified as actions while nouns like “security” and “profit” and adjectives like “safe” and “familiar” are classified as concepts. As concepts describe what and how things are, the higher the conceptualization, the higher the knowledge.

## Results and discussion

### Citizen participation in ELAs

Table 2 reports standardized OLS regressions of the number of ELAs on several sociodemographic and political variables. Number of ELAs, Population, and Number of Organizations are count variables, and Mayor, Incumbent Mayor, and SUBDERE groups are categorical. The remaining variables are expressed as the share of the population. For instance, Rurality represents the share of the municipality-level population living in rural areas. Variables with high skewness have been transformed with a base 10 log function. The transformed variables are Number of ELAs, Population, Population Density, and Number of Community Organizations. The p-value of the Ramsey Regression Equation Specification Error Test (RESET) is provided in the caption of each table throughout this work. Only significant interactions are shown in Table 2, but the full list of interactions can be found in the S6 Table in S1 File.

**Table 2:** OLS regressions. p-value RESET test Model 1 = 0.109, p-value RESET test Model 2 = 0.1418, p-value RESET test Model 3 = 0.3501. RESET test were performed on the second power of regressors.

	*Outcome variable*:		
	log (1 + ELAs)		
	(1)	(2)	(3)
Log (population)	0.528 **	0.718 **	0.778 **
	(0.131)	(0.142)	(0.164)
Higher Education	0.156 **	0.128 *	0.185 **
	(0.047)	(0.050)	(0.065)
Internet penetration rate	0.113 **	0.105 *	0.103 **
	(0.036)	(0.041)	(0.039)
*SUBDERE groups* ^(1)^ (control)	yes	yes	yes
Log (community organizations)	0.103 *	0.059	0.085
	(0.048)	(0.056)	(0.051)
Born in 1981 or after		-0.032	-0.008
		(0.052)	(0.056)
Rurality		-0.081	-0.052
		(0.056)	(0.054)
Log (population density)		-0.100 *	-0.139 **
		(0.050)	(0.053)
Women		0.067	0.066
		(0.072)	(0.069)
Two-parent family (with children)		-0.133 **	-0.093 *
		(0.040)	(0.043)
Single-parent family (with children)		-0.065	-0.101 *
		(0.041)	(0.043)
Votes for current president			0.148 **
			(0.046)
Municipal officials			-0.040
			(0.170)
Voter turnout			0.087
			(0.054)
Mayor (government)			0.063
			(0.069)
Mayor (opposition)			-0.099
			(0.079)
Party affiliation			0.161 *
			(0.066)
Incumbent Mayor (True)			-0.020
			(0.054)
Evangelical Christians			-0.075 **
			(0.027)
Constant	-0.072	0.042	0.037
	(0.192)	(0.203)	(0.209)
Observations	313	313	310
R^2^	0.784	0.804	0.834
Adjusted R^2^	0.777	0.790	0.814
Residual Std. Error	0.463 (df = 301)	0.449 (df = 291)	0.424 (df = 275)
F Statistic	99.586**	56.800**	40.652***
F Statistic	(df = 11; 301)	(df = 21; 291)	(df = 34; 275)

Note:

*p<0.05;

**p<0.01

^(1)^ SUBDERE groups are a socio-demographic classification based on county’s dependence on the municipal common fund.

^(2)^ Only significant interactions are shown.

The number of ELAs strongly depends on the population. As expected, this variable is the most variance-explicative predictor of citizen participation.

Model 1 Table 2 includes variables for socioeconomic and social capital. This model shows significant and positive effects for higher education, internet penetration rate, and the number of community organizations. According to the literature on political participation and social capital, education has a high impact both on social participation [75] and in the understanding of complex political information [16]. As expected, our results confirm a significant and positive return to education (measured as the share of people with higher education) on the number of ELAs. Furthermore, from all the significant regressors of the full model, Education has the second-highest coefficient, after Population (Table 2).

Internet Penetration rate shows a consistent, positive, and significant effect on the number of ELAs. This result is consistent with previous research showing a positive impact of the Internet on social relations and civic engagement. However, this variable has been derived from a CASEN survey that is representative only of 139 of the 346 municipalities in Chile. To assess the real impact of CASEN’s lack of representativeness on the Internet penetration variable, we compared the following: (i) full model with all the 328 counties comprised in CASEN survey, (ii) full model with the 139 counties where the CASEN survey is representative, and (iii) bootstrapping of the full model with random samples of 139 counties (The actual number of observations in each case is slightly different due to missing values.) We used (iii) to evaluate whether the changes from (i) to (ii) resulted from the reduction of the sample size. The result of this comparison is shown in S12 Table in S1 File. We show that when considering only the 139 counties for which the CASEN survey is representative, the effect of the Internet penetration rate becomes non-significant. However, in the bootstrapped regression the effect is also non-significant and the error increases, suggesting that the loss of statistical significance may be due to the smaller sample.

The socioeconomic factor, represented here by the SUBDERE groups, shows no significant effect in the number of ELAs. SUBDERE classification reflects the municipality’s dependence on the Municipal Common Fund, and it is used here as a proxy for municipal residents’ incomes. Since the sociodemographic groups created by SUBDERE incorporate the population in the municipality classification, this variable may be correlated with population density. Therefore, Model 3 in Table 2 (henceforth called “the full model”) was repeated, replacing SUBDERE classification with a poverty measure, which also shows no significant effect (S9 Table in S1 File). The regression results using Poverty are shown in S9 Table of S1 File. Replacing SUBDERE with Poverty in the model does not substantially change the effect of the other socioeconomic or sociodemographic variables.

Model 1 Table 2 also includes the number of community organizations in our model as a proxy for social capital at the municipality level. The result shows a positive but non-significant effect, suggesting that this variable can be correlated to another sociopolitical variable or that the number of community organizations does not represent actual participation in those organizations.

Model 2 Table 2 includes Population Density and Two-parent Family (with Children) as demographic variables that show significant and negative effects. The negative effect of population density is in agreement with previous research reporting that high population density has a negative impact on social capital [22]. Regression results show a positive effect on the share of women and a negative effect on the youngest age cohort. Even though both effects are non-significant, their signs are in agreement with voting behavior in Chile, where the younger cohorts exhibit the lowest participation rate, and voter turnout rates for women exceed those for men [12]. Our model also indicates that fewer ELAs were organized in municipalities with a higher proportion of single-parent families and two-parent families with children. An intuitive explanation is that people with children have less time to engage in social activities. However, previous research has reported a negative effect of having children in female labor force participation, which in turn, has a negatively impact on their social capital and political participation [11].

Finally, Model 3 incorporates political variables (For a performance plot, see S3 Fig in S1 File.) The share of votes going to the winning candidate in the first round of the presidential election has a positive and significant effect on participation. This effect remains when considering the voting of the second ballot (S10 Table in S1 File). This result suggests that people who supported the winning candidate were more willing to organize and participate in an ELA since the whole idea of the new constitution was devised and started by the government they chose. On the other hand, voter turnout for the first round of the 2013 presidential election had a positive but non-significant effect in the number of ELAs (Table 2). For the runoff election, however, the effect became positive and significant (S10 Table in S1 File). Note that voter turnout for the second round was about seven percentage points lower than for the first round (50% in the first round, 43% in the runoff ballot), which is not unusual. The phenomenon of turnout decline in runoff elections has been studied for the past 40 years. In particular, the decline is higher when the election result seems predetermined, and when the vote proportion received in the first round by candidates not qualifying for the runoff goes up [76, 77]. The latter implies that the runoff election does not involve the diversity of ideas that led many voters to turn out at the first ballot. In simple words, many of those who abstain from the second ballot do so because no candidate represents them. Therefore, the positive and significant effect of voter turnout in the runoff election may also reflect citizen support for the winning candidate.

Party affiliation, when considered as a variable including parties both within the government coalition and within the opposition coalition, is a positive and significant variable (Table 2). When the model incorporates only parties within the government coalition or only parties within the opposition coalition, the effect remains positive and significant. This suggests that people more actively engaged in politics have a better disposition to participate in an ELA, even if they dislike the government proposing the constitution-making idea. We also explored political mobilization by evaluating the political affiliation of the mayor. No significant effect was found. To test whether mobilization was driven by members of Parliament, we created a new variable we named Government Influence that combines the political affiliation of mayors and deputies. In S11 Table in S1 File, the variables Mayor and Incumbent Mayor were replaced with this variable, but this effect was not significant.

Finally, the negative and significant effect of the share of Evangelical Christians on the number of ELAs presents a question on the role of religion in society. It has been argued that churches in the United States play an important role in building up the civic skills of those otherwise least likely to participate in politics [55]. This might not be true for Chilean evangelicals, who often perceive themselves as second-class citizens legally and socially disadvantaged in comparison to the members of the Catholic Church [78] and who therefore might be inclined to disengage from political activity.

As for robustness checks, we note that the variance inflation factors are all less than 5 except for Education (5.5) and Population (10.3) (S5 Table in S1 File). On the other hand, considering that the dependent variable in Eq 1 is a count variable, we ran a negative binomial regression, which accounted for over-dispersion (see S7 Table in S1 File). Comparison with Model 3 Table 2 shows no significant changes for the main results. Therefore, all subsequent models were estimated using OLS.

### Topic modeling and text analysis

We analyzed the argument texts using STM because that tool enables the estimating of topics conditioned to document-level metadata. The corpus consisted of all argument texts that were written for Constitutional Rights throughout all ELAs. Each text corresponds to a document of the corpus, and the constitutional concept to which the text refers was incorporated as prevalence document metadata.

We estimated an STM with 23 topics in our corpus. We chose the number of topics by performing various model diagnostics such as Held-Out Likelihood and the Semantic Coherence for a different number of topics (See S4 Fig in S1 File.) We also included the constitutional rights concepts as prevalence covariates in the model, and the authors manually assigned the topic labels by looking at two sets of words for each topic. The first is a set of highest probability words inferred directly from topic-word distribution. The second is a set of words that are both frequent and exclusive for each topic [79]. Both sets of words can be found in S5 and S6 Figs of S1 File.

Table 3 shows both sets of words for four different topics (Education, Equality, Security, and Environment). These topics were selected as examples because they cover a broad range of themes and show considerable differences in content when comparing groups across different determinants.

**Table 3:** OLS regressions results for STM. Table shows the top three categories for each regression. Concepts in italic font were not included in the original list of concepts proposed by the government, and were added by ELAs participants. The last two columns in the table show the word sets for three topics. The words shown here have been translated into English. All the STM analysis has been performed with the original texts in Spanish.

Topic	Right	*Outcome variable*:	Highest Probability	Frequent and exclusive
		Topic		
Education	Education	0.336(0.004)***	education, quality, for-free, must, access, universal, public, level, free education, (there must) be education, profit, public education, secular, integral, free-of-charge, public for-free, higher, opportunity, civic	for-free secular, integral education, quality for-free, university, teacher, room, public free education, free education, free-of-charge, public education, higher education, (there must) be education, must guarantee education, decent education, (there must) be free education, guarantee education, student, free access
	*Right to quality public health care*	0.092(0.014)***		
	Freedom of Education	0.091(0.007)***		
Equality	Equality before the law	0.345(0.005)***	equality, law, must, same, to-exist, justice, (there must) be equality, opportunity, must exist, treatment, access, same right, process, privilege, gender, to-treat, can, egalitarian	must exist difference, same condition, due process, have equality, military, exist difference, (there must) be equality, justice, same right, equal treatment, equality, law, to-exist equality, privilege, must exist equality, same treatment, to-exist privilege, same opportunity, must have equality
	Access to justice / due process	0.286(0.009)***		
	Equality	0.258(0.005)***		
Security	Security / non-violence	0.358(0.005)***	to-live, must, violence, security, safe, can, peace, space, to-feel, quiet, get-better, can live, crime, home, house, fear, tranquility, (there must) be security	(there must) be greater, to-feel, must live, insecurity, neighborhood, crime, (there must) be protection, peace, violence, can live, (there must) be security, quiet, street, safe, tranquility, to-live quietly
	Freedom of movement	0.112(0.019)***		
	Decent housing	0.089(0.004)***		
Environment	Environmental respect / protection	0.468(0.008)***	must, environment, resource, good, natural, better, nature, natural resource, to-live, water, generation, pollution, free, use, future, to-preserve, respect, sustainable	ecosystem, environment free, better quality, pollution, future generation, healthy environment, environment, better, water, good, must preserve, clean, natural resource, nature, sustainable, sustainability, sustainable development
	*Right to water*	0.295(0.023)***		
	*Conservation of cultural and historical heritage*	0.147(0.055)		

*** *p* < 0.001,

** *p* < 0.01,

* *p* < 0.05

We then considered how each constitutional concept affects the topic proportion. We ran a regression model in which each document is an observation; the outcome variable is the probability that a specific topic generates each document in the STM model (Remember that we model each document as a weighted combination of topics.), and the explanatory variables are all constitutional rights. Therefore, the estimated coefficients represent, for each topic, the effect of the constitutional rights on the topic. Table 3 shows the regression results for the same four topics. Since each topic has 59 explanation variables (the total number of constitutional rights), we display here only the top three categories, (i.e., the constitutional concepts with the highest topic proportion) within each topic, for each regression. The results show agreement between the topics emerging from the argument texts and the concepts that originated those texts. The regression results for the remaining topics can be found in S13 Table of S1 File.

**Fig 2:**
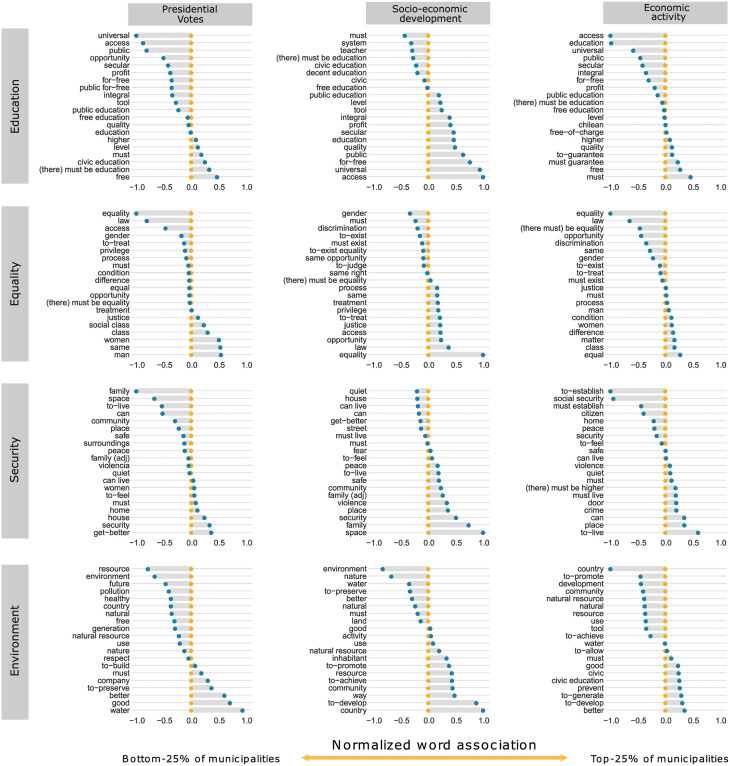
A word comparison of the constitutional rights debate, at the municipality level. We show the emergent topics: Education, Equality, Security, and Environment for three different citizen participation determinants: presidential votes, socioeconomic development, and economic activity. Words are oriented along the X-axis based on how much they are associated to the inspected determinant. For instance, in the Education topic and Presidential Votes determinant, the word “universal,” which is on the left side, is associated with the municipalities where the elected president got the least votes. The word “free,” which is on the right side, is associated with the municipalities where the elected president got most of the votes. We note that for the topic Equality, the word “process” comes from “due process” and “treatment” refers to “behaviour towards.” Likewise, for the topic Security, “door” comes from “revolving door,” which refers to inmate release and recidivism, and “be able to live” comes from “be able to live peacefully in our neighborhood.”

How do the sociopolitical determinants of citizen participation affect topics discussions? To address this question, we estimated a second STM model with prevalence and topic content covariates. Recalling that, while topic prevalence captures how much each topic contributes to a document, topic content variables enable the vocabulary used to discuss a particular topic to vary. Here, we use three different determinants as covariates: Presidential Votes, Socioeconomic Development, and Primary Economic Activity. For each variable, we compared the municipalities in the top and bottom quartiles. Fig 2 shows the differences in vocabulary by association level with the determinants for those same four topics: Education, Equality, Security, and Environment. The list of words for each group and topic along with representative phrases can be found in S14–S17 Tables in S1 File. In the case of the topic Environment, the word “water” is strongly associated with the top government supporting left-wing municipalities, municipalities with low population density, and those with a lower proportion of highly educated people (S7 Fig in S1 File). This is also related to the concept *Right to Water* that emerged during the ELAs, and supports the nationalization of natural resources. On the other hand, the topic Education shows a transverse support for free and public education, while words like “access” and “universal” predominate in highly educated, more socioeconomically developed, and right-wing municipalities. For topic Equality, highly educated, more socioeconomically developed, and right-wing municipalities discuss “law” and “equality” more, while the opposite groups are more concerned about “gender equality” and “social classes.”

Topic modeling also enables us to explore the emergent content of the whole corpus. Given that each document is associated with a particular constitutional concept, the STM shows us how different constitutional concepts mix into topics. For instance, the topic Equality (Table 3) contains three related concepts of similar proportions (*Equality Before the Law, Access to Justice*, and *Equality*). This mixture of topics is especially relevant to many of the concepts added by the participants, even though they were already variations or combinations of existing concepts. For example, the topic Environment comprises the *Right to Water*, which was not included in the original list. Fig 2 shows that the word “water,” probably arising from the *Right to Water*, is strongly associated with a political position. It is worth noting that the concept *Freedom of Movement* is associated with the topic Security, suggesting that this right is interpreted as “moving safely” rather than the right to travel from place to place or to change the place where one resides or works.

Finally, the terms shown in Fig 2 can also be categorized according to their linguistic usage, (i.e. according to a content/style classification). We used the same four topics to assess the difference in argument quality, but focused only on Socioeconomic Development and Primary Economic Activity determinants because the presidential vote may illuminate political differences in language usage but is unlikely to directly affect the quality of the deliberation, which is our main interest in this final section.

For each topic, we extracted the argument texts from the top three rights related to that topic (See Table 3). Then we compared the proportion of texts that were classified as low-quality arguments within the municipalities of the top and bottom quartiles (See Table 4 and S8 Fig in S1 File.) We performed this comparison using a two-sample test for equality of proportions (*χ*^2^ test). For example, for a given topic and covariate, we estimated the proportion of high quality arguments for that topic between the top 25 percent and the bottom 25 percent of municipalities on that covariate. We found no significant differences for the topic Security, but for the topics Environment and Education, both variables showed significant differences: for the topic Environment, 53% of texts were classified as high quality for the top quartile of Socioeconomic Development, versus 40% for the bottom quartile, and 34% of texts were classified as high quality for the top quartile of Primary Economic Activity versus 53% for the bottom quartile. For topic Education 53% of texts were classified as high quality for the top quartile of Socioeconomic Development, versus 46% for the bottom quartile, and 46% of texts were classified as high quality for the top quartile of Primary Economic Activity versus 54% for the bottom quartile. For topic Equality, only the socioeconomic development shows significant difference (49% of high quality text for the top quartile versus %41 for the bottom quartile). The percentages reported here were estimated with a 2-terms search. Table 4 shows the list of terms, and the p-values for a 2-terms and 3-terms search.

**Table 4:** p-values, Chi-Square test for differences in the proportion of the argument quality.

Topic	Variable	p-value (2 terms)	p-value (3 terms)	List of terms
Equality	SEDI	0.02	0.05	igualitario, sentencia, normas jurídicas, trato igualitario, diferencia, justicia, mismas normas, proceso judicial, género, condición, político, existir persona, debe indemnizar, oportunidad, afectación grave, misma forma, ley, juicio justo, privilegio, mismas oportunidades, mismos derechos, mismas leyes, proceso, igualdad, condiciones, acceso, carga público, mismo derecho, ley acceso, inocencia, proceso justo, judicial, mismo trato, debido proceso, grupo privilegiado, rico.
	Prim. Econ. Act.	0.16	0.31	
Security	SEDI	0.76	0.15	caminar, tranquilidad, calle, comunidad, barrio, seguridad, espacio, tranquilo, preso, casa, debe haber política, vivir, paz, inseguridad, seguridad ciudadana, violencia, país seguro, lugar, delincuente, haber protección, sentir, seguro, seguridad personal, ciudad, policía, miedo, delincuencia, hogar.
	Prim. Econ. Act.	0.64	0.55	
Education	SEDI	0.04	<0.01	cívica, nivel, sala, oportunidad, formación, superior, públic, preescolar, universal, lucro, secundaria, sala cuna, universitaria, conocimiento, inclusiva, laica, acceso, gratis, gratuita, integral, calidad, igualitaria.
	Prim. Econ. Act.	0.01	<0.01	
Environment	SEDI	0.01	0.06	aire, renovable, fauna, mejor sociedad, ambiente libre, contaminación, sustentable, natural, futuras generaciones, energía, planeta, ecosistema, futuro, conservación, desarrollo, ambiente limpio, entorno, generación, ambiente sano, flora, alimento, ambiente saludable, sustentabilidad, recurso, agua.
	Prim. Econ. Act.	<0.01	<0.01	

For the topics Environment and Equality, we found that municipalities with a higher socioeconomic development index produce higher quality arguments (S8 Fig in S1 File). Also, these municipalities use more concepts than their less developed peers, particularly for topics Equality and Security, whereas the municipalities with a higher share of people engaging in primary economic activity use more for the topic Security as well. It is therefore hypothesized that conceptualization is related to knowledge. Knowledge facilitates proposing themes, concepts, and ideas. Therefore, at the municipality level, the greater the knowledge, the greater the conceptualization. Furthermore, the use of verbal forms such as “There must be,” and “must have,” are linked to normative or prescriptive intentions. In many of our cases, the terms classified as actions are actually this type of verbal form.

Now let us take a look at the topic Security in the context of the Socioeconomic Development variable (Fig 2). The four actions most associated with the less developed municipalities are “be able to live,” “can,” “must live,” and “must.” This indicates a predominantly normative intention from this group. However, a sentiment analysis also reveals that these terms score higher (positively) when compared to the more developed municipalities (S18 Table in S1 File). This is mainly caused by the predominant use of verbs by this group. In general, verbs in this corpus have a positive connotation: “get-better,” “guarantee,” and “be able to live,” for example. All of this suggests that this normative intention may be driven by the sentiment that if the matter concerns you, you probably won’t produce conceptually dense arguments; rather, you will express a desire and a proposal for action.

## Conclusions

The Chilean constitution-making process of 2015–2016 was a unique experiment in terms of the political history of the country and of the unprecedented level of participation and territory coverage when compared to similar processes held in other countries [6]. Our results (Table 2) show that engagement in politics and support for the government increased participation. However, no evidence of political mobilization by mayors or deputies was found, suggesting that citizen involvement in the constitutional process was not ideologically biased but rather voluntarily biased. As the OECD report conjectures, “Those citizens who support the acting government may be more likely to participate in the consultation, even when all citizens are given that opportunity” [6]. On the other hand, when using a structural topic modeling approach, we found that the emerging content from ELAs can be mapped ideologically.

Although the Chilean constitution-making process reached a high level of citizen participation, this does not necessarily imply it generated high-quality public deliberation. Our analysis suggests that the high-quality argumentation came from the municipalities with the highest level of socioeconomic development (S8 Fig in S1 File). Moreover, the STM results show that people in municipality with high levels of socioeconomic development and comparatively more complex economic structures (both proxies for knowledge) use more themes, concepts and ideas than actions when deliberating, suggesting that knowledge facilitates conceptualization. Finally, our citizen participation model also indicates (as is widely known in political science) that education increases political participation. In sum, these results point to the Public Ignorance Objection, an important criticism of deliberative democracy, that claims that the educated citizens of a society have the most effective tools to deliberate. Such groups resemble “epistemic communities” [80, 81], for they can provide information and advice, becoming actors in political decision-making processes.

Today’s discussion is not about the pros and cons of participatory democracy, but rather the extent of its expression and how we can improve its function [37]. Along with the theoretical discussion about deliberative democracy, there is a good deal of research on deliberative citizen forums [82], that informs about good practices in deliberative processes. For example, regarding group composition, when participants confront their out-group (i.e., with people who hold an opposite view in a particular issue), they are more likely to hold more positive out-group attitudes after the experiment, regardless of the quality of the deliberation [83]. There is also an effect of public perceptions of legitimacy due to the selection mechanism (sortition compared to election) and participants’ profile (lay citizens versus professional politicians) [84]. However, neither legitimacy nor representativeness can assure a good quality deliberation. Other features to consider are the use of moderators and a set of rules for discussion [85], and the information provided to participants before the discussion [86], among others.

The OECD has recently summarized some of these lessons in a “good practice” guideline for deliberative processes [87]. These recommendations include different aspects, such as privacy, representativeness, inclusiveness, and transparency, among others. Regarding group deliberation, they suggest “a mix of formats that alternate between small group and plenary discussions and activities, and skilled facilitation” [85].

The Chilean processes exhibited at least two critical design weaknesses: (i) the voluntary nature of the encounters increased participation biases, thus diminishing representativeness, and (ii) information asymmetry between groups, resulting in a difference in deliberation quality. Nonetheless, the participative phase of the Chilean constitution-making process still played an essential role in gathering information and visualizing people’s needs. Beyond the quality of the deliberation, citizen participation—when adequately designed—impacts itself. On the citizens, participation would have an “emancipatory effect,” where symbolic perspectives before deliberation give space to a broader range of considerations on the issue [37]. There is also an increase in legitimacy, which—although it is difficult to measure—would positively impact the acceptance of norms and institutions, thus benefiting society as a whole. Indeed, the result of the deliberation—and consequently, the political process on which the participative process is part—will depend on the quality of the deliberation. Therefore, it is crucial to design the process carefully and to have tools and metrics to evaluate the process performance.

Our results can inform the design of new massive deliberative consulting processes, which can be convenient for some types of political endeavors. Future research should explore the complexity of deliberative discussions in different socio-cultural contexts. Thus, to evaluate how the deliberative process can generate solutions for complex problems. Finally, new ways to scale designs of citizen consultations for political participation should be a matter of new research and development [88].

## Supporting information

S1 Dataset (CSV)

S2 Dataset (CSV)

S1 File (PDF)
