# Supplementary material for: Citizens at the forefront of the constitutional debate: Voluntary citizen participation determinants and emergent content in Chile
Source: PLoS One. 2022 Jun 6;17(6):e0267443. doi: 10.1371/journal.pone.0267443 (PMC9170096; doi:10.1371/journal.pone.0267443)
Supplement: S1 File — (PDF) [file pone.0267443.s003.pdf]

Supplementary Information

Citizens at the forefront of the constitutional debate: Voluntary citizen participation determinants and emergent content in Chile

Age and gender

Table S1: Contingency table for participants in ELAs and national population, by age cohorts. Pearson's Chi-squared test results for age cohorts: X-squared = 78.989, p-value $< 2.2 \cdot 10^{-6}$

	Born in 1980 or after	Born between 1956 and 1980	Born in 1955 or before
National population	6146410	5656007	2483653
Participants in ELAs	46078	39902	18216

Table S2: Contingency table for participants in ELAs and national population, by gender. Pearson's Chi-squared test results for gender : X-squared = 277.61, p-value $< 2.2 \cdot 10^{-6}$

	Female	Male
National population	7361978	6924092
Participants in ELAs	56393	47803

Statistical model: Size of ELAs

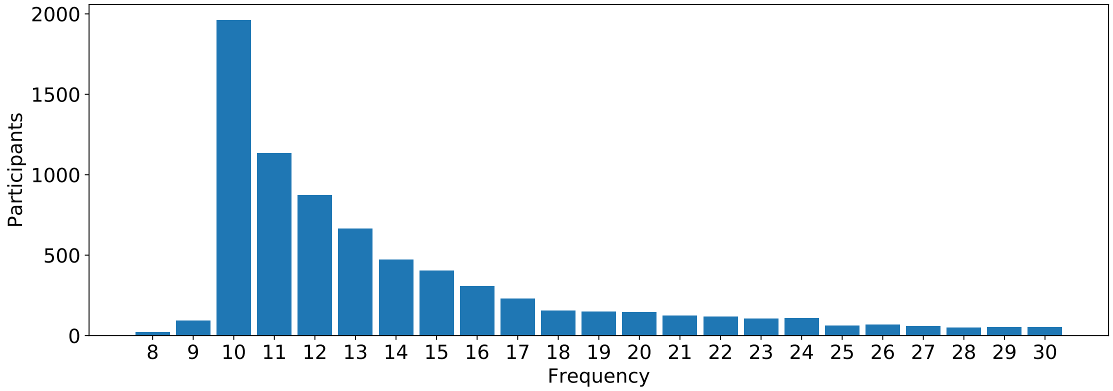

Figure S1: Histogram of ELAs number of participants.

ELAs : Questions and concepts.

What should be the fundamental and universal RIGHTS contained in the Constitution? Choose up to seven topics among the list below or suggest others in the free space.

Table S3: ELAs : Questions and concepts.

Original concepts:	
Suffrage/vote	Honor/reputation
Nationality	Right of association
Election to public office	Peaceful assembly
Participation	Request before the authorities
Life	Freedom to work
Mental and physical integrity	Freedom of Education
Security/non-violence	Right to Work
Equality	Fair wage
Non-discrimination	Decent housing
Equality before the law	Healthcare
Access to justice/due process	Education
Equality in relation to public burdens	Social security
Tax equality	Right to organize and to collective bargaining
Gender equity	Right to strike
Children's and teenager's rights	Access to culture
Integration of disabled people	Cultural identity
Personal freedom	Indigenous people
Freedom of movement	Environmental respect/protection
Freedom of conscience	Property
Freedom of expression	Judicial protection of individual rights
Right to information	Free economic initiative/free enterprise
Access to public information	None
Privacy and intimacy	Others, specify
New concepts:	
Standard of living	Cultural identity of indigenous people
	Freedom of worship
Respect life from conception	Right to water
Right to make one's own decisions about one's life	Freedom
Right to work and a decent wage	Human Rights
Social Rights	Freedom of information and speech
Animal rights	Conservation of cultural and historical heritage

Table S4: Descriptive statistics

Statistic	N	Mean	St. Dev.	Min	Max
Number of ELAs	345	22.31	52.65	0	530
Number of participants	345	302.02	705.62	0	7,233
Population	345	41,408.90	64,527.83	230	453,530
Population density	345	953.44	2,989.08	0.03	17,144.86
Rurality	345	0.36	0.29	0.00	1.00
Higher education	345	0.19	0.10	0.06	0.76
Number of organizations	342	472.99	599.02	3.00	4,892.00
Participation in comm. org.	324	0.07	0.08	0.0000	0.65
Internet penetration rate	324	0.05	0.12	−0.20	0.90
SEDI	324	0.54	0.12	0.24	0.99
Poverty	344	0.13	0.08	0.001	0.42
Born in 1981 or after	345	0.40	0.05	0.29	0.60
Men	345	0.51	0.06	0.44	0.86
Women	345	0.49	0.06	0.14	0.56
Single-parent family with children	345	0.12	0.02	0.01	0.17
Two-parent family with children	345	0.28	0.05	0.07	0.45
Voter turnout	345	0.50	0.08	0.15	0.68
Votes for current president	345	0.52	0.09	0.13	0.73
Voter turnout (runoff)	345	0.43	0.08	0.10	0.63
Votes for current president (runoff)	345	0.64	0.09	0.18	0.82
Party affiliation	345	0.08	0.07	0.01	0.94
Municipal officials	345	0.01	0.01	0.001	0.09
Evangelical Christians	341	0.13	0.08	0.01	0.49

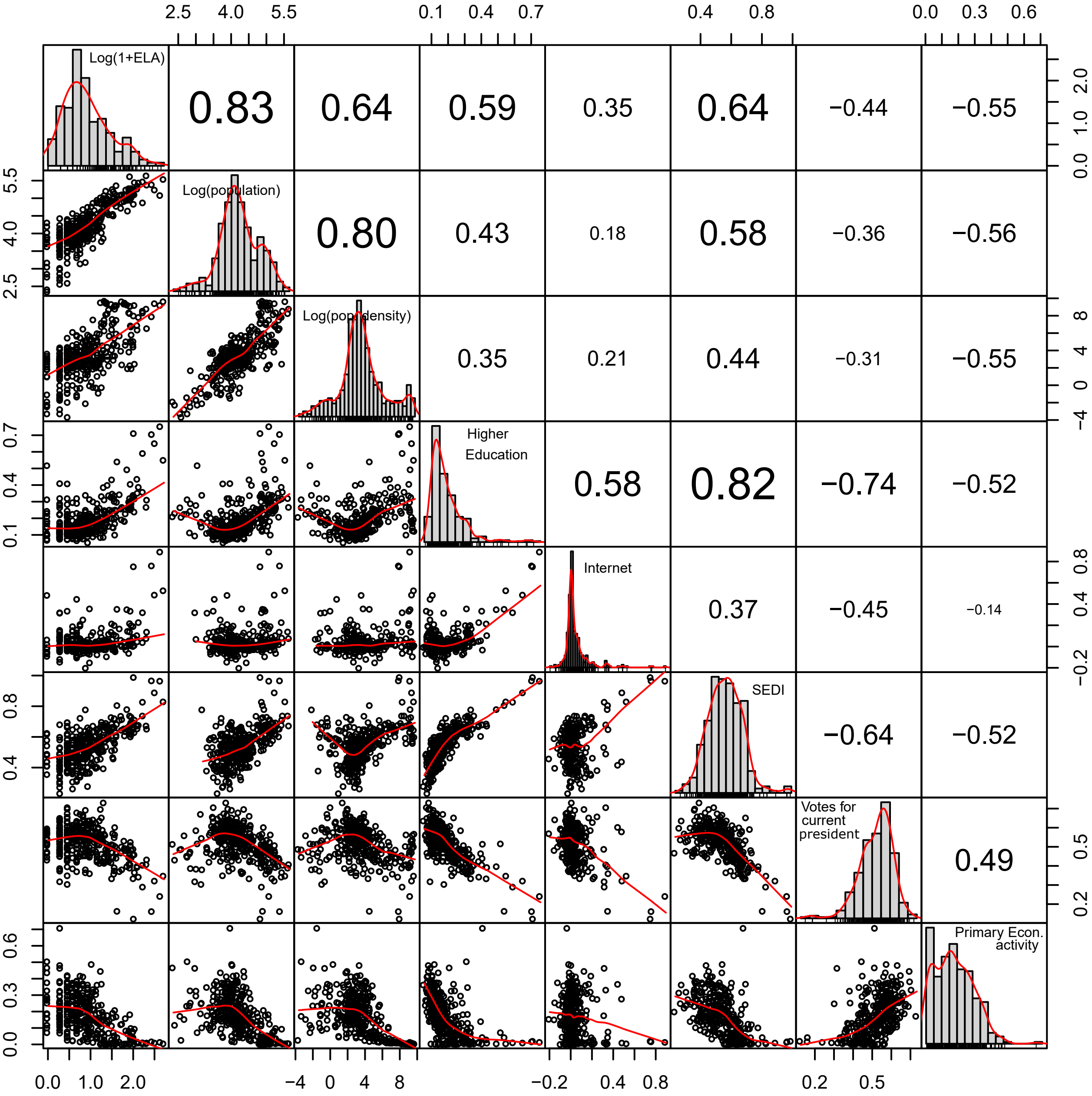

Figure S2: Correlogram of main variables. All Pearson correlations are significant at the $p < 0.05$ significance level.

Results

Table S5: Variance inflation factor (VIF) for predictors in OLS model.

Regressor	VIF
Log (population)	10.3908
Higher education	5.5627
Internet penetration rate	2.0860
Log (community organizations)	3.0870
Born in 1981 or after	3.0040
Rurality	3.0160
Log (population density)	3.4063
Women	2.5619
Two-parent family (with children)	1.9531
Single-parent family (with children)	1.4367
Votes for current president	2.9716
Municipal officials	3.1048
Voter turnout	2.4574
Party affiliation	1.7249
Evangelical Christians	1.2018

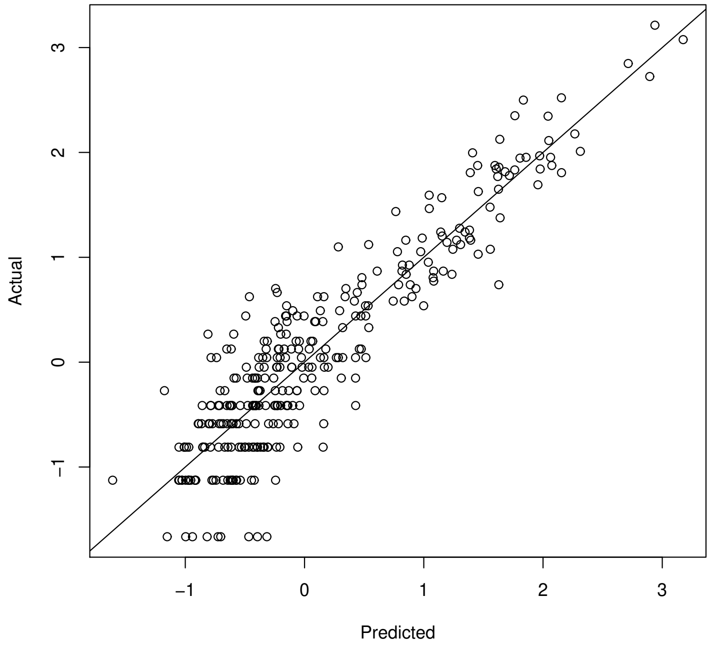

Figure S3: Actual versus Predicted values for the number of ELAs, model (3) of Table 2.

Table S6: OLS estimates for the standardized full model with robust standard errors, p-value RESET test = 0.3501. RESET test were performed on the second power of regressors.

	Dependent variable:
	log (1 + ELAs)
Log (population)	0.778 (0.164)**
Higher education	0.185 (0.065)**
Internet penetration rate	0.103 (0.039)**
SUBDERE groups	
Group 2	−0.122 (0.154)
Group 3	−0.234 (0.196)
Group 4	−0.129 (0.224)
Group 5	0.016 (0.277)
Group 6	0.297 (0.351)
Group 7	0.497 (0.417)
Group 8	0.236 (0.421)
Log (community organizations)	0.085 (0.051)
Born in 1981 or after	−0.008 (0.056)
Rurality	−0.052 (0.054)
Log (population density)	−0.139 (0.053)**
Women	0.066 (0.069)
Two-parent family (with children)	−0.093 (0.043)*
Single-parent family (with children)	−0.101 (0.043)*
Votes for current president	0.148 (0.046)**
Municipal officials	−0.040 (0.170)
Voter turnout	0.087 (0.054)
Mayor (government)	0.063 (0.069)
Mayor (opposition)	−0.099 (0.079)
Party affiliation	0.161 (0.066)*
Incumbent mayor (True)	−0.020 (0.054)
Evangelical Christians	−0.075 (0.027)**
Rurality * Evangelical Christians	0.040 (0.049)
Log (population density) * Evangelical Christians	−0.055 (0.067)
Log (community organizations) * Born in 1981 or after	−0.054 (0.043)
Born in 1981 or after * Internet penetration rate	0.005 (0.018)
Rurality * Log (community organizations)	−0.053 (0.050)
Log (population density) * Log (community organizations)	−0.054 (0.041)
Municipal officials * Voter turnout	0.039 (0.095)
Municipal officials * Mayor (government)	0.204 (0.181)
Municipal officials * Mayor (opposition)	−0.027 (0.184)
Constant	0.037(0.209)
Observations	310
Adjusted R^2	0.814
Residual Std. Error	0.424 (df = 275)
F Statistic	40.652** (df = 34; 275)

Note: $^{*}p<0.05$; $^{**}p<0.01$. The base categories for dummy variables are: "Group 1" for SUBDERE groups, "False" for Incumbent Mayor an "Independent" for Mayor. See Appendix Table Al for more detailed variable definitions and sources

Table S7: OLS and Negative Binomial estimates for the full model.

	Dependent variable:	
	OLS	Negative binomial
	log (1 + ELAs)	ELAs
Log(population)	0.778**	1.263**
	(0.164)	(0.218)
Higher education	0.185**	0.282**
	(0.065)	(0.099)
Internet penetration rate	0.103**	0.116*
	(0.039)	(0.054)
SUBDERE groups		
Group 2	−0.122	−0.221
	(0.154)	(0.285)
Group 3	−0.234	−0.403
	(0.196)	(0.336)
Group 4	−0.129	−0.327
	(0.224)	(0.368)
Group 5	0.016	−0.212
	(0.277)	(0.421)
Group 6	0.297	0.038
	(0.351)	(0.507)
Group 7	0.497	0.222
	(0.417)	(0.570)
Group 8	0.236	−0.142
	(0.421)	(0.572)
Log (community organizations)	0.085	0.128
	(0.051)	(0.077)
Born in 1981 or after	−0.008	−0.072
	(0.056)	(0.085)
Rurality	−0.052	−0.119
	(0.054)	(0.083)
Log (population density)	−0.139**	−0.238**
	(0.053)	(0.073)
Women	0.066	0.045
	(0.069)	(0.098)
Two-parent family (with children)	−0.093*	−0.135*
	(0.043)	(0.059)
Single-parent family (with children)	−0.101*	−0.146*
	(0.043)	(0.065)
Votes for current president	0.148**	0.231**
	(0.046)	(0.065)
Municipal officials	−0.040	0.008
	(0.170)	(0.279)
Voter turnout	0.087	0.135
	(0.054)	(0.090)
Mayor (government)	0.063	0.072
	(0.069)	(0.108)
Mayor (opposition)	−0.099	−0.211
	(0.079)	(0.129)
Party affiliation	0.161*	0.239*
	(0.066)	(0.096)
Incumbent mayor (True)	−0.020	−0.004
	(0.054)	(0.073)
Evangelical Christians	−0.075**	−0.084*
	(0.027)	(0.041)
Constant	0.037	2.308***
	(0.209)	(0.340)
Observations	310	310
Adjusted R^2	0.814	
Log Likelihood		-930.405
θ		6.338** (0.893)
Akaike Inf. Crit.		1,930.810
Residual Std. Error	0.424 (df = 275)	
F Statistic	40.652** (df = 34; 275)	

Note: *p<0.05; **p<0.01. The base categories for dummy variables are: "Group 1" for SUBDERE groups, "False" for Incumbent Mayor an "Independent" for Mayor. Only significant interactions are shown. For both models, all independent variables have been standardized. The dependent variable (*ELAs*) is also standardize in the OLS model, whereas it is kept as a count variable in the Negative Binomial model.

Table S8: OLS estimates for the number of ELAs (log (1 + ELAs), p-value RESET test = 0.3501) and the number of participants (log (1 + participants), p-value RESET test = 0.4672). RESET tests were performed on the second power of regressors.

	Outcome variable:	
	log (1 + ELAs)	log (1 + participants)
Log(population)	0.778^{**}	0.747^{**}
	(0.164)	(0.181)
Higher education	0.185^{**}	0.148^{*}
	(0.065)	(0.073)
Internet penetration rate	0.103^{**}	0.114^{*}
	(0.039)	(0.045)
SUBDERE groups		
Group 2	−0.122	−0.067
	(0.154)	(0.210)
Group 3	−0.234	−0.153
	(0.196)	(0.261)
Group 4	−0.129	0.020
	(0.224)	(0.290)
Group 5	0.016	0.154
	(0.277)	(0.352)
Group 6	0.297	0.250
	(0.351)	(0.439)
Group 7	0.497	0.385
	(0.417)	(0.500)
Group 8	0.236	0.044
	(0.421)	(0.501)
Log (community organizations)	0.085	0.103
	(0.051)	(0.061)
Born in 1981 or after	−0.008	0.041
	(0.056)	(0.066)
Rurality	−0.052	0.014
	(0.054)	(0.061)
Log(population density)	-0.139^{**}	−0.076
	(0.053)	(0.063)
Women	0.066	0.017
	(0.069)	(0.095)
Two-parent family (with children)	-0.093^{*}	-0.100^{*}
	(0.043)	(0.050)
Single-parent family (with children)	-0.101^{*}	−0.078
	(0.043)	(0.053)
Votes for current president	0.148^{**}	0.135^{*}
	(0.046)	(0.056)
Municipal officials	−0.040	0.024
	(0.170)	(0.194)
Voter turnout	0.087	0.178^{*}
	(0.054)	(0.075)
Mayor (government)	0.063	0.095
	(0.069)	(0.088)
Mayor (opposition)	−0.099	−0.136
	(0.079)	(0.100)
Party affiliation	0.161^{*}	0.200^{*}
	(0.066)	(0.078)
Incumbent mayor (True)	−0.020	−0.005
	(0.054)	(0.067)
Evangelical Christians	-0.075^{**}	-0.071^{*}
	(0.027)	(0.033)
Constant	0.037	0.008
	(0.209)	(0.272)
Observations	310	310
Adjusted R^2	0.814	0.698
Residual Std. Error (df = 275)	0.424	0.511
F Statistic (df = 34; 275)	40.652^{**}	22.046^{**}

Note: $^{*}p<0.05$; $^{**}p<0.01$. The base categories for dummy variables are: "Group 1" for SUBDERE groups, "False" for Incumbent Mayor an "Independent" for Mayor. Only significant interactions are shown.

Table S9: OLS estimates for the full model (p-value RESET test = 0.3501), and replacing *SUBDERE groups* with *poverty* (p-value RESET test = 0.2828). RESET tests were performed on the second power of regressors.

	Outcome variable:	
	log (1 + ELAs)	
	SUBDERE Groups	Poverty
Log(population)	0.778**	0.971**
	(0.164)	(0.112)
Higher education	0.185**	0.248**
	(0.065)	(0.071)
Internet penetration rate	0.103**	0.071
	(0.039)	(0.043)
SUBDERE groups		
Group 2	−0.122	
	(0.154)	
Group 3	−0.234	
	(0.196)	
Group 4	−0.129	
	(0.224)	
Group 5	0.016	
	(0.277)	
Group 6	0.297	
	(0.351)	
Group 7	0.497	
	(0.417)	
Group 8	0.236	
	(0.421)	
Poverty		0.033
		(0.041)
Log (community organizations)	0.085	0.085
	(0.051)	(0.051)
Born in 1981 or after	−0.008	0.003
	(0.056)	(0.047)
Rurality	−0.052	−0.028
	(0.054)	(0.056)
Log (population density)	−0.139**	−0.124*
	(0.053)	(0.052)
Women	0.066	0.077
	(0.069)	(0.073)
Two-parent family (with children)	−0.093*	−0.106*
	(0.043)	(0.042)
Single-parent family (with children)	−0.101*	−0.094*
	(0.043)	(0.048)
Votes for current president	0.148**	0.153**
	(0.046)	(0.045)
Municipal officials	−0.040	0.013
	(0.170)	(0.146)
Voter turnout	0.087	0.065
	(0.054)	(0.055)
Mayor (government)	0.063	0.120
	(0.069)	(0.068)
Mayor (opposition)	−0.099	−0.036
	(0.079)	(0.078)
Party affiliation	0.161*	0.217**
	(0.066)	(0.068)
Incumbent mayor (True)	−0.020	−0.013
	(0.054)	(0.052)
Evangelical Christians	−0.075**	−0.087**
	(0.027)	(0.031)
Constant	0.037	−0.043
	(0.209)	(0.055)
Observations	310	320
Adjusted R^2	0.814	0.810
Residual Std. Error	0.424 (df = 275)	0.432 (df = 291)
F Statistic	40.652** (df = 34; 275)	49.414** (df = 28; 291)

Note: *p<0.05; **p<0.01. The base categories for dummy variables are: "Group 1" for SUBDERE groups, "False" for Incumbent Mayor an "Independent" for Mayor. Only significant interactions are shown.

Table S10: OLS estimates for the full model (p-value RESET test = 0.3501) and replacing the votes for the incumbent president and the voter turnout with the corresponding runoff variables (p-value RESET test = 0.4605). RESET tests were performed on the second power of regressors.

	Dependent variable:	
	log (1 + ELAs)	
	First round	Runoff
Log(population)	0.778^{**}	0.763^{**}
	(0.164)	(0.168)
Higher education	0.185^{**}	0.131^{*}
	(0.065)	(0.060)
Internet penetration rate	0.103^{**}	0.111^{**}
	(0.039)	(0.039)
SUBDERE groups		
Group 2	−0.122	−0.123
	(0.154)	(0.155)
Group 3	−0.234	−0.236
	(0.196)	(0.197)
Group 4	−0.129	−0.115
	(0.224)	(0.229)
Group 5	0.016	0.043
	(0.277)	(0.282)
Group 6	0.297	0.352
	(0.351)	(0.361)
Group 7	0.497	0.567
	(0.417)	(0.429)
Group 8	0.236	0.297
	(0.421)	(0.428)
Log (community organizations)	0.085	0.085
	(0.051)	(0.051)
Born in 1981 or after	−0.008	0.006
	(0.056)	(0.061)
Rurality	−0.052	−0.048
	(0.054)	(0.055)
Log (population density)	-0.139^{**}	-0.175^{**}
	(0.053)	(0.053)
Women	0.066	0.065
	(0.069)	(0.070)
Two-parent family (with children)	-0.093^{*}	-0.110^{*}
	(0.043)	(0.046)
Single-parent family (with children)	-0.101^{*}	-0.098^{*}
	(0.043)	(0.043)
Votes for current president	0.148^{**}	
	(0.046)	
Votes for current president (runoff)		0.089^{*}
		(0.038)
Municipal officials	−0.040	−0.043
	(0.170)	(0.170)
Voter turnout	0.087	
	(0.054)	
Voter turnout (runoff)		0.155^{*}
		(0.060)
Mayor (government)	0.063	0.064
	(0.069)	(0.069)
Mayor (opposition)	−0.099	−0.117
	(0.079)	(0.080)
Party affiliation	0.161^{*}	0.161^{*}
	(0.066)	(0.067)
Incumbent mayor (True)	−0.020	−0.022
	(0.054)	(0.054)
Evangelical Christians	-0.075^{**}	-0.074^{**}
	(0.027)	(0.028)
Constant	0.037	0.016
	(0.209)	(0.213)
Observations	310	310
Adjusted R^2	0.814	0.813
Residual Std. Error (df = 275)	0.424	0.425
F Statistic (df = 34; 275)	40.652^{**}	40.479^{**}

Note: $^{*}p<0.05$; $^{**}p<0.01$. The base categories for dummy variables are: "Group 1" for SUBDERE groups, "False" for Incumbent Mayor an "Independent" for Mayor. Only significant interactions are shown.

Table S11: OLS estimates for the full model (p-value RESET test = 0.3501), and replacing the variables Mayor and Incumbent Mayor with a governments' influence variable (p-value RESET test = 0.6196). RESET tests were performed on the second power of regressors.

	Dependent variable:	
	log (1 + ELAs)	
	Mayor	Gov. Influence
Log(population)	0.778^{**}	0.775^{**}
	(0.164)	(0.158)
Higher education	0.185^{**}	0.195^{**}
	(0.065)	(0.062)
Internet penetration rate	0.103^{**}	0.097^{*}
	(0.039)	(0.039)
SUBDERE groups		
Group 2	−0.122	−0.119
	(0.154)	(0.159)
Group 3	−0.234	−0.222
	(0.196)	(0.198)
Group 4	−0.129	−0.131
	(0.224)	(0.226)
Group 5	0.016	0.009
	(0.277)	(0.276)
Group 6	0.297	0.305
	(0.351)	(0.344)
Group 7	0.497	0.496
	(0.417)	(0.408)
Group 8	0.236	0.238
	(0.421)	(0.408)
Log (community organizations)	0.085	0.083
	(0.051)	(0.050)
Born in 1981 or after	−0.008	−0.012
	(0.056)	(0.056)
Rurality	−0.052	−0.065
	(0.054)	(0.053)
Log (population density)	-0.139^{**}	-0.143^{**}
	(0.053)	(0.053)
Women	0.066	0.055
	(0.069)	(0.071)
Two-parent family (with children)	-0.093^{*}	-0.099^{*}
	(0.043)	(0.044)
Single-parent family (with children)	-0.101^{*}	-0.097^{*}
	(0.043)	(0.043)
Votes for current president	0.148^{**}	0.159^{**}
	(0.046)	(0.045)
Municipal officials	−0.040	−0.005
	(0.170)	(0.149)
Voter turnout	0.087	0.077
	(0.054)	(0.053)
Mayor (government)	0.063	
	(0.069)	
Mayor (opposition)	−0.099	
	(0.079)	
Gov. influence		0.027
		(0.032)
Party affiliation	0.161^{*}	0.161^{*}
	(0.066)	(0.068)
Incumbent mayor (True)	−0.020	
	(0.054)	
Evangelical Christians	-0.075^{**}	-0.069^{*}
	(0.027)	(0.027)
Constant	0.037	0.020
	(0.209)	(0.206)
Observations	310	310
R^2	0.834	0.831
Adjusted R^2	0.814	0.813
Residual Std. Error	0.424 (df = 275)	0.425 (df = 278)
F Statistic	40.652^{**} (df = 34; 275)	44.234^{**} (df = 31; 278)

Note: *p<0.05; **p<0.01. The base categories for dummy variables are: "Group 1" for SUBDERE groups, "False" for Incumbent Mayor an "Independent" for Mayor. Only significant interactions are shown.

Table S12: OLS estimates for the full model (p-value RESET test = 0.3501) using all the municipalities, and the municipalities where CASEN is representative (139 municipalities). The third column shows the result of a bootstrap of 139 samples.

	Dependent variable:		
	log (1 + ELAs)		
	Full sample	CASEN sample	Bootstrap
Log(population)	0.778**	1.045**	0.815**
	(0.164)	(0.218)	(0.247)
Higher education	0.185**	0.284*	0.209*
	(0.065)	(0.124)	(0.102)
Internet penetration rate	0.103**	0.009	0.086
	(0.039)	(0.072)	(0.061)
SUBDERE groups			
Group 2	−0.122	−1.254**	−0.137
	(0.154)	(0.446)	(0.248)
Group 3	−0.234	−1.058**	−0.263
	(0.196)	(0.370)	(0.300)
Group 4	−0.129	−1.215**	−0.156
	(0.224)	(0.422)	(0.348)
Group 5	0.016	−1.102*	−0.050
	(0.277)	(0.472)	(0.422)
Group 6	0.297	−1.153*	0.219
	(0.351)	(0.570)	(0.542)
Group 7	0.497	−1.227	0.405
	(0.417)	(0.628)	(0.652)
Group 8	0.236	−1.458	0.177
	(0.421)	(0.745)	(0.790)
Log (community organizations)	0.085	−0.010	0.075
	(0.051)	(0.093)	(0.089)
Born in 1981 or after	−0.008	0.205	−0.025
	(0.056)	(0.119)	(0.085)
Rurality	−0.052	−0.076	−0.042
	(0.054)	(0.139)	(0.090)
Log (population density)	−0.139**	−0.233*	−0.114
	(0.053)	(0.102)	(0.105)
Women	0.066	0.236	0.012
	(0.069)	(0.269)	(0.144)
Two-parent family (with children)	−0.093*	−0.229**	−0.094
	(0.043)	(0.072)	(0.068)
Single-parent family (with children)	−0.101*	−0.270*	−0.057
	(0.043)	(0.107)	(0.100)
Votes for current president	0.148**	0.201**	0.125
	(0.046)	(0.075)	(0.130)
Municipal officials	−0.040	0.111	0.015
	(0.170)	(0.398)	(0.275)
Voter turnout	0.087	0.196	0.096
	(0.054)	(0.112)	(0.094)
Mayor (government)	0.063	−0.137	0.020
	(0.069)	(0.155)	(0.125)
Mayor (opposition)	−0.099	−0.523*	−0.084
	(0.079)	(0.248)	(0.169)
Party affiliation	0.161*	0.118	0.192
	(0.066)	(0.172)	(0.142)
Incumbent mayor (True)	−0.020	0.140	−0.013
	(0.054)	(0.080)	(0.078)
Evangelical Christians	−0.075**	−0.017	−0.071
	(0.027)	(0.064)	(0.048)
Municipal officials * Voter turnout	0.039	0.453*	0.067
	(0.095)	(0.224)	(0.206)
Constant	0.037	1.092**	0.087
	(0.209)	(0.418)	(0.327)
Observations	310	132	
Adjusted R^2	0.814	0.853	
Residual Std. Error	0.424 (df = 275)	0.370 (df = 97)	
F Statistic	40.652** (df = 34; 275)	23.431** (df = 34; 97)	

Note: *p<0.05; **p<0.01. The base categories for dummy variables are: "Group 1" for SUBDERE groups, "False" for Incumbent Mayor an "Independent" for Mayor. Only significant interactions are shown.

Table S13: OLS regressions results for STM. Table shows the top three categories for each regression. Concepts in italic font were not included in the original list of concepts proposed by the government and were added by ELAs participants.

		Outcome variable: Topic
Topic 1: Environment	Environmental respect / protection	0.468 (0.008)**
	Right to water	0.295 (0.023)**
	Conservation of cultural and historical heritage	0.147 (0.055)
Topic 2: Life	*Respect life from conception*	0.401 (0.047)**
	Life	0.265 (0.004)**
	Mental and physical integrity	0.164 (0.007)**
Topic 3: Public policy	*Animal rights*	0.409 (0.044)**
	Privacy and intimacy	0.100 (0.012)**
	Right of association	0.099 (0.011)**
Topic 4: Unclassified	Election to public office	0.102 (0.015)**
	Decent housing	0.097 (0.004)**
	Freedom to work	0.094 (0.013)**
Topic 5: Non - discrimination	Non - discrimination	0.293 (0.008)**
	Gender equity	0.105 (0.006)**
	Equality	0.101 (0.004)**
Topic 6: Development	Free economic initiative / free enterprise	0.209 (0.013)**
	Human Rights	0.171 (0.014)**
	Property	0.142 (0.009)**
Topic 7: Participation	Participation	0.457 (0.012)**
	Suffrage / vote	0.424 (0.011)**
	Election to public office	0.407 (0.023)**
Topic 8: Rights	Judicial protection of individual rights	0.204 (0.008)**
	Respect life from conception	0.189 (0.032)**
	Life	0.145 (0.003)**
Topic 9: Security	Security / non-violence	0.358 (0.005)**
	Freedom of movement	0.112 (0.019)**
	Decent housing	0.089 (0.004)**
Topic 10: Education	Education	0.336 (0.004)**
	Right to quality public health care	0.092 (0.014)**
	Freedom of Education	0.091 (0.007)**
Topic 11: Equality before the law	Equality before the law	0.345 (0.005)**
	Access to justice / due process	0.286 (0.009)**
	Equality	0.258 (0.005)**
Topic 12: Social security	Social security	0.261 (0.005)**
	Decent housing	0.257 (0.008)**
	Right to work and a decent wage	0.148 (0.014)
Topic 13: Unclassified	Tax equality	0.437 (0.027)**
	Equality in relation to public burdens	0.194 (0.042)**
	Request before the authorities	0.112 (0.022)**
Topic 14: Indigenous people	Indigenous people	0.520 (0.011)**
	Cultural identity of indigenous people	0.506 (0.060)**
	Cultural identity	0.471 (0.017)**
Topic 15: Labor rights	Right to organize and to collective bargaining	0.413 (0.009)**
	Right to strike	0.378 (0.013)**
	Right to work and a decent wage	0.302 (0.016)**
Topic 16: Freedom of education	Freedom of Education	0.437 (0.012)**
	Free economic initiative / free enterprise	0.304 (0.022)**
	Property	0.286 (0.012)**
Topic 17: Integration	Integration of disabled people	0.243 (0.009)**
	Equality before the law	0.150 (0.005)**
	Non - discrimination	0.110 (0.004)**
Topic 18: Childhood	Children and teenager's rights	0.295 (0.007)**
	Human Rights	0.141 (0.012)**
	Judicial protection of individual rights	0.132 (0.009)**
Topic 19: Social rights	*Social rights*	0.184 (0.009)**
	Social security	0.129 (0.005)*
	Standard of living	0.117 (0.008)
Topic 20: Healthcare	Healthcare	0.296 (0.006)**
	Right to quality public health care	0.273 (0.018)**
	Access to public information	0.194 (0.011)**
Topic 21: Freedom	*Freedom of worship*	0.551 (0.017)**
	Freedom of information and speech	0.435 (0.039)**
	Freedom of expression	0.411 (0.006)**
Topic 22: Fair wage	Fair wage	0.261 (0.007)**
	Gender equity	0.155 (0.007)**
	Freedom of worship	0.128 (0.016)**
Topic 23: Unclassified	*Conservation of cultural and historical heritage*	0.090 (0.039)
	Healthcare	0.081 (0.003)**
	Right to quality public health care	0.080 (0.009)**

$^{**}p < 0.01$, $^{*}p < 0.05$

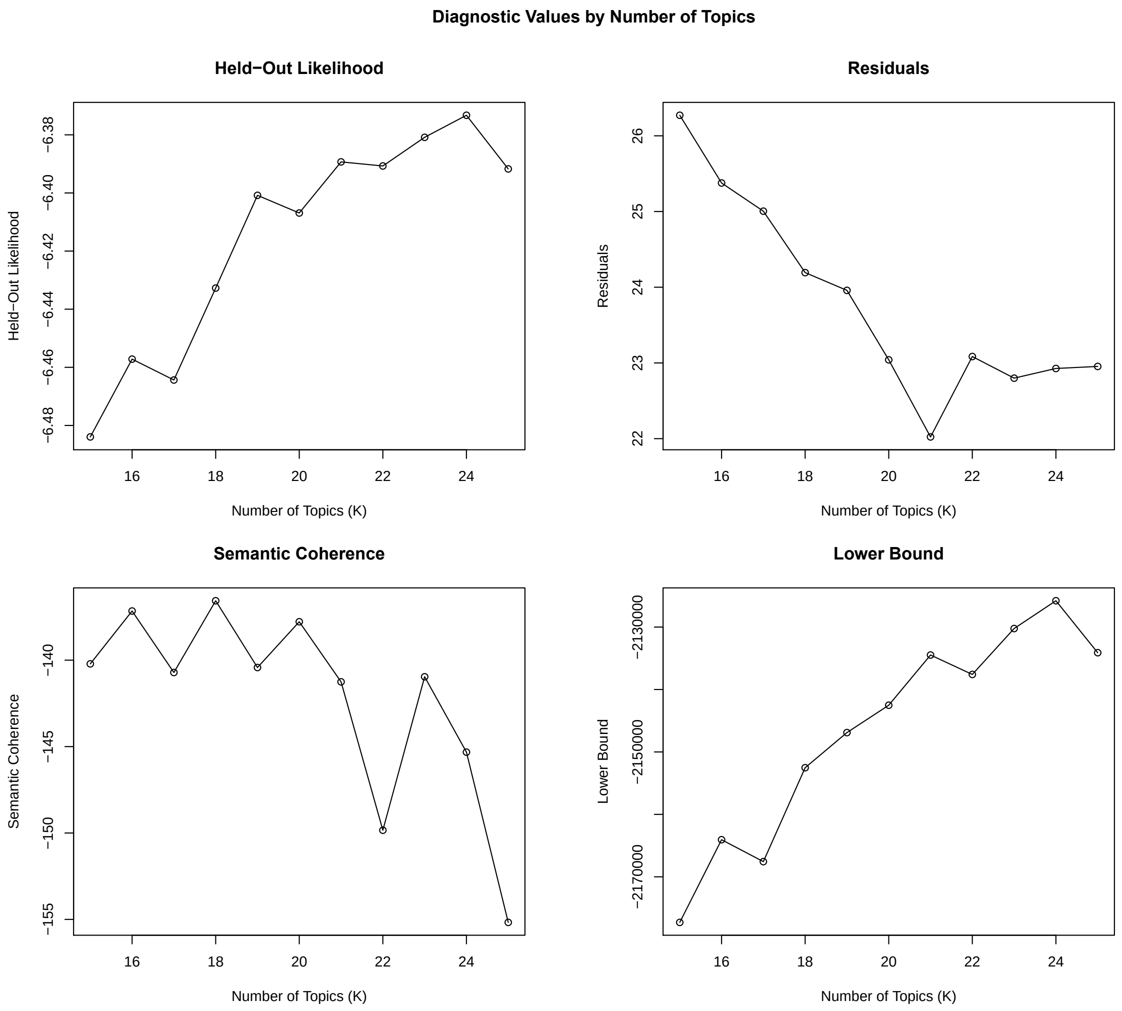

Figure S4: Diagnostic values by number of topics. The optimal number of topics should seek to maximize the Held-Out Likelihood (top-left panel) and the Semantic Coherence (bottom-left panel), and minimize the residual dispersion (top-right panel). For 23 topics, both the Held-Out Likelihood and the Semantic Coherence are reasonably close to their maximum values, while the residual dispersion has reached a stationary value (see (Wallach et al. 2009; Taddy 2012; Mimno et al. 2011) for further information on these indicators).

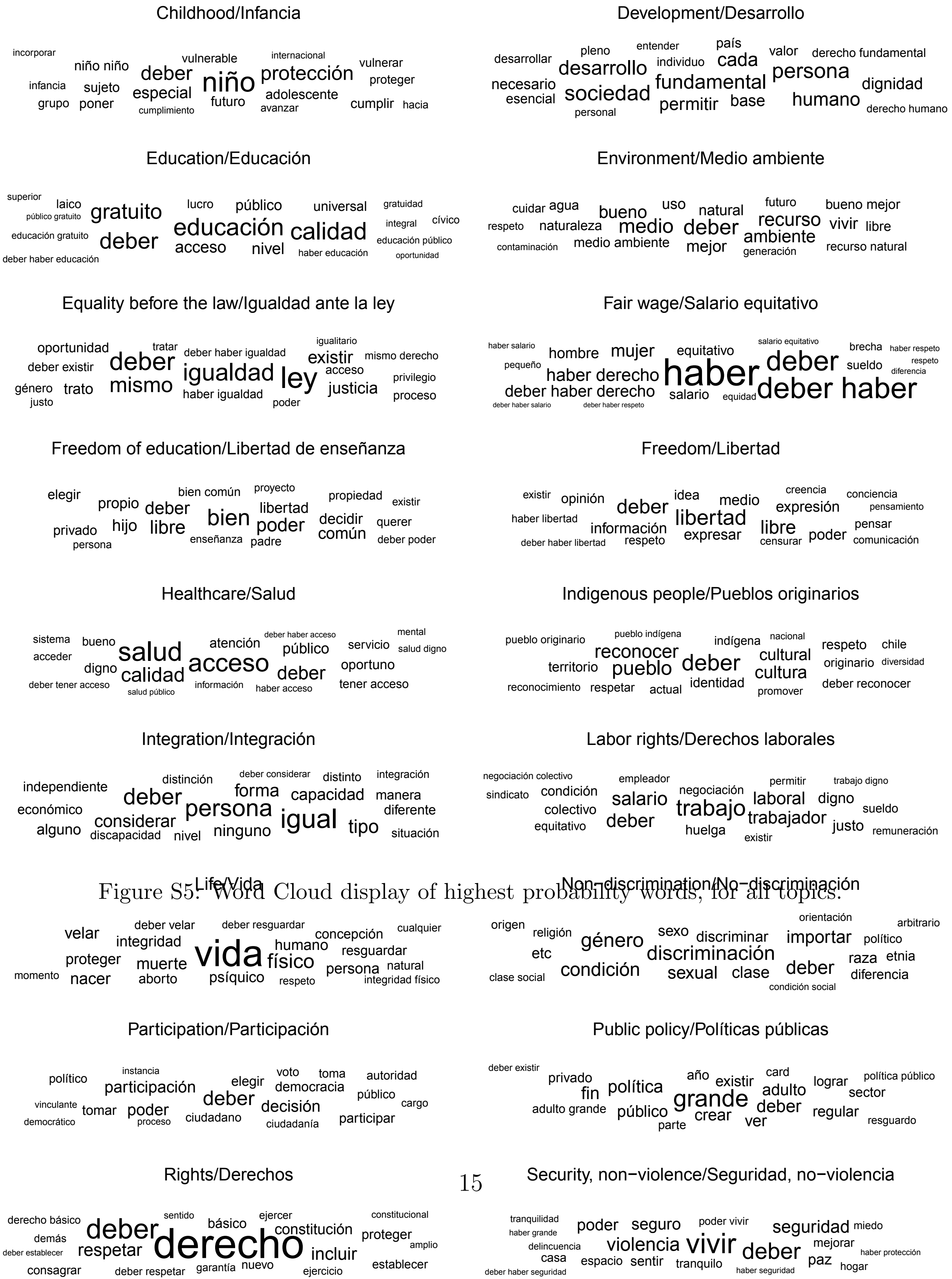

Figure S5: Word Cloud display of highest probability words, for all topics.

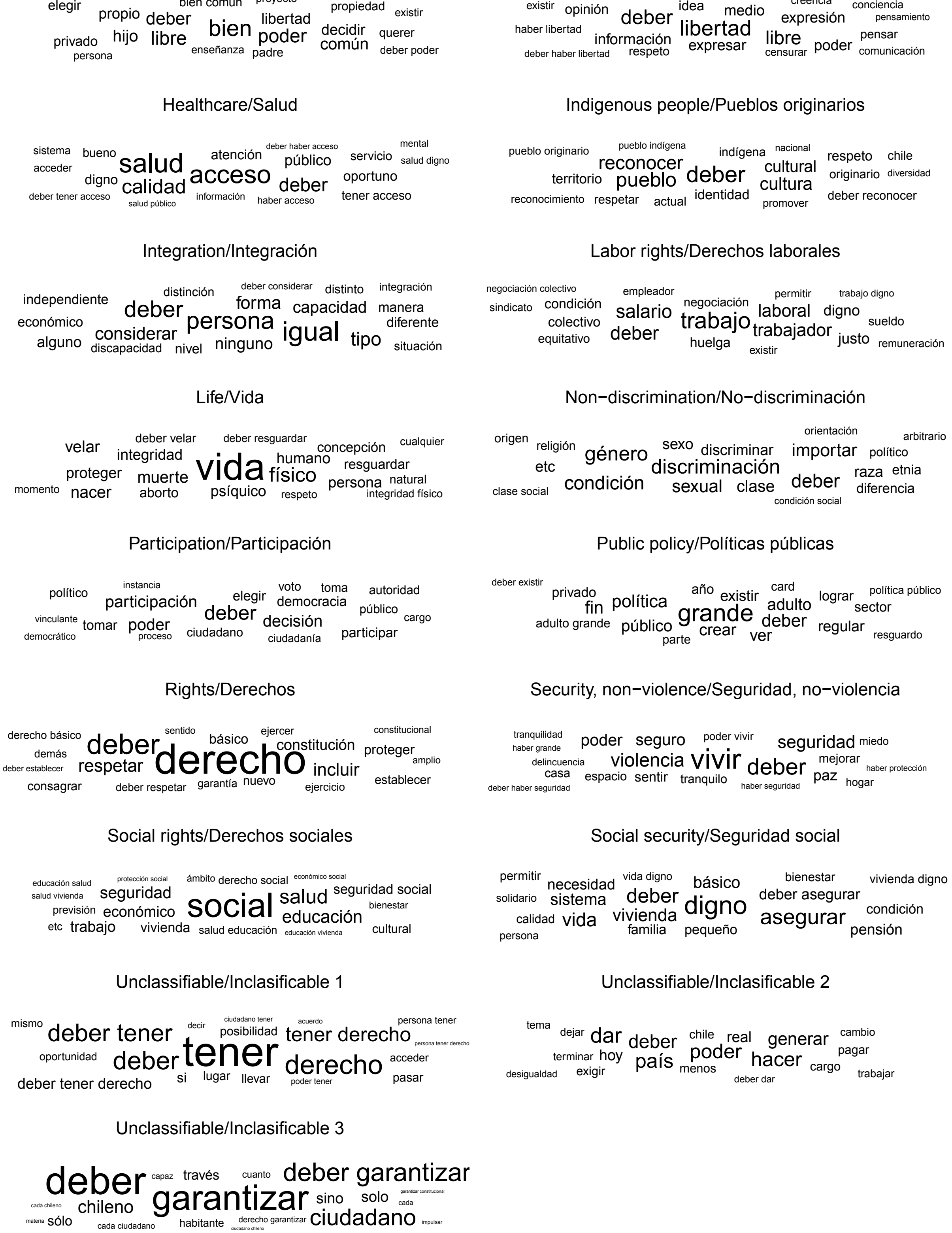

Word Cloud display of highest probability words, for all topics (continuation of Fig. S5).

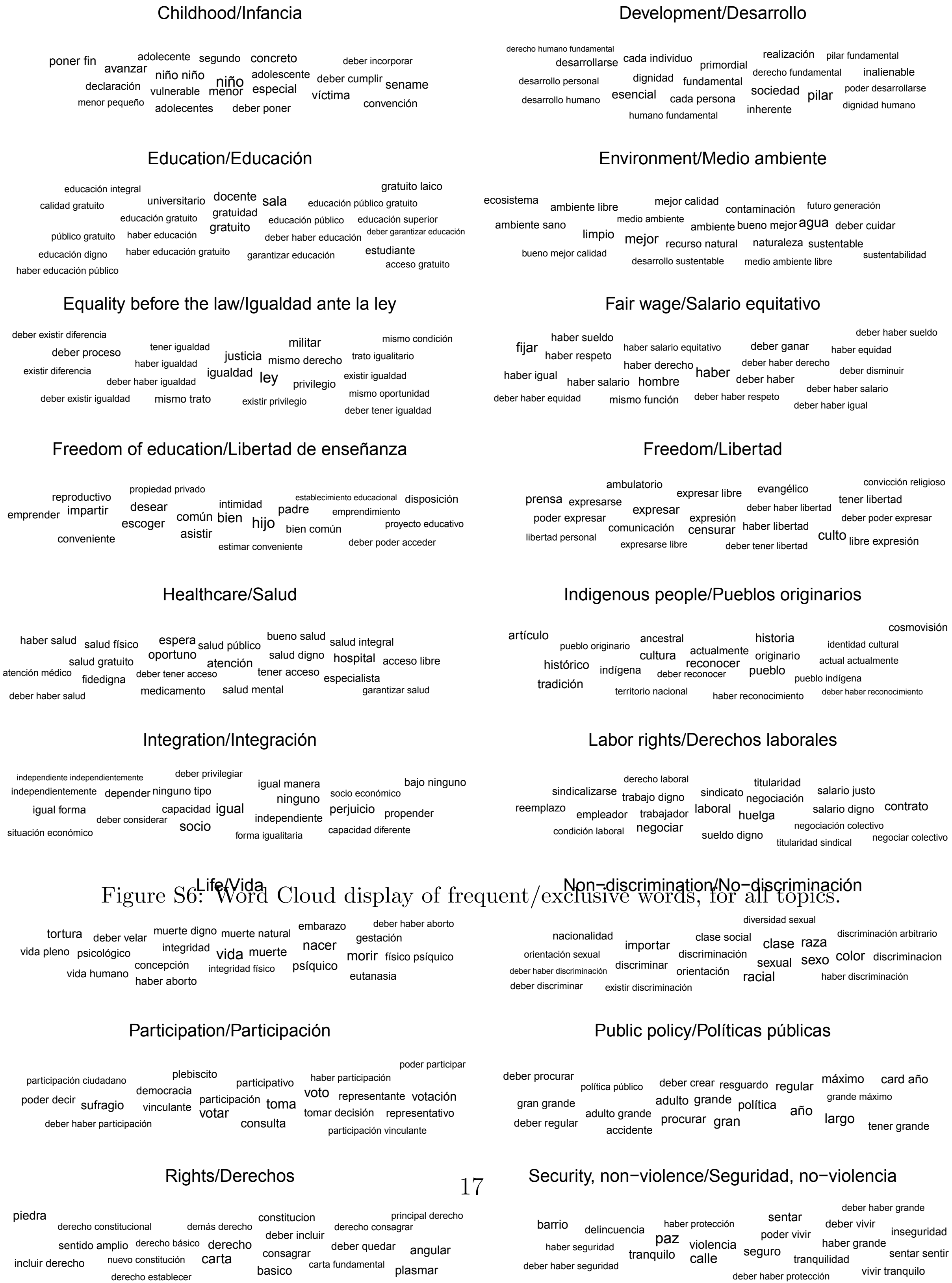

Figure S6: Word Cloud display of frequent/exclusive words, for all topics.

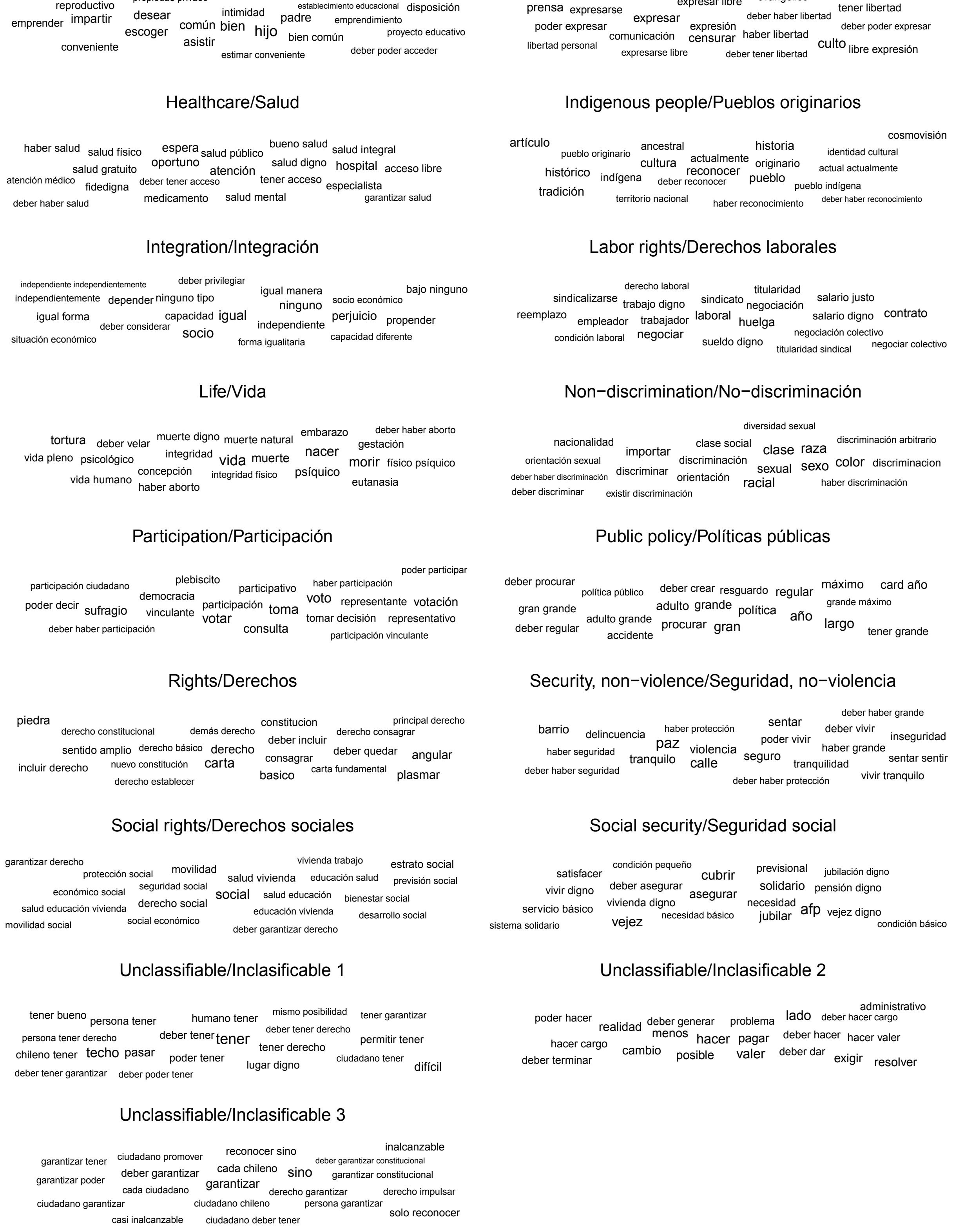

Word Cloud display of frequent/exclusive words, for all topics (continuation of Fig. S6).

Table S14: Topic: Environment

Determinant	Quartile	Topic Words	City	Text
Votes	Bottom 25	recurso, medio ambiente, futuro, contaminación, sano, país, natural, libre, generación, recurso natural, uso, naturaleza, respeto.	Concón	Debe poder incentivar la creación de empresas entregando empleo y generando desarrollo.
			Vitacura	Debe haber derecho a la vida en un medio ambiente libre de contaminación.
			Lo Barnechea	Es fundamental para lograr tener una buena calidad de vida lo cual se logra a través del respeto de a la naturaleza y medio ambiente siendo labor de todos cuidarlo
	Top 25	construir, deber, empresa, cuidar, mejor, bueno, agua.	Canela	Tenemos derecho a disfrutar y tener acceso a los recursos naturales y cuidarlo para las futuras generaciones.
			Puyehue	Debe asegurar recursos como son el agua aire tierra suelo y sub suelo.
			La Pintana	Debemos mantenernos en equilibrio con la naturaleza para que las nuevas generaciones tenga un hábitat mejor pues se ha demostrado que gran parte de las enfermdades y catástrofes son responsabilidad de nosotros y las grandes empresas.
Higher education	Bottom 25	agua, deber, mejor, bueno, actividad, tierra, naturaleza.	Combarbabá	Debe nacionalizar y distribuir el agua equitativamente regresando al estado y no siguiendo en manos de los privados que lucran sin medida con ella ya que sin agua no hay vida.
			Placilla	Debe dejar un mejor país a los hijos y nietos educando a los habitantes para que protejan la naturaleza.
			Collipulli	Debe tener un plan de contignecia ante las sequias que son comunes en nuestra región y localidad en específico habiendo derecho al agua para su consumo producción y sus animales.
	Top 25	cuidar, libre, cuidado, contaminación, generación, recurso natural, natural, futuro, recurso, sano, uso, medio ambiente, país.	Valdivia	Debe asegurar el respeto y cuidado por el medio ambiente en este derecho descansa el cuidado de nuestros recursos naturales.
			Valparaíso	Debe cuidar medio ambiente ya que la naturaleza forma parte de nuestras vidas responsables
			Antofagasta	Debemos conservar nuestro planeta sano.
Pop. density	Bottom 25	agua, deber, respeto, país, cuidado, uso.	Chile Chico	Debe garantizar el respeto y protección del medio ambiente y la naturaleza.
			Tierra Amarilla	Debe cuidar aguas dulces sin contaminarlas.
			Pozo Almonte	Debe haber respeto a todo lo que hace posible la vida en el planeta y a las condiciones para que pueda seguir existiendo la vida humana.
	Top 25	naturaleza, cuidar, contaminación, libre, fomentar, recurso natural, recurso, generación, natural, futuro, medio ambiente, sano, mejor, bueno.	Viña del Mar	Debe haber cuidado de la naturaleza preservando lo que existe y legislando para mejorar la protección del medio ambiente.
			Puente Alto	Debemos mantener el medio ambiente para las generaciones futuras.
			Talcahuano	Debe tener derecho a vivir en ambiente sano y un medio ambiente limpio.
SEDI	Bottom 25	ambiente, naturaleza, agua, cuidar, mejor, natural, deber ,tierra.	Monte Patria	Debe tener ambiente mejor y más limpio para el futuro.
			Chanco	Debe cuidar de nuestros bienes naturales en la medida que no son renovables y cautelar la sobreexplotación.
			San Nicolás	Debe cuidar el medio ambiente pero de verdad con leyes que protejan la naturaleza sin distinción.
	Top 25	bueno, actividad, uso, recurso natural, habitante, fomentar, recurso, lograr, comunidad, manera, desarrollar, país.	Calama	Deben ser usados administrados y explotados para el provecho de las comunidades en que se encuentran y del país como nación.
			Las Condes	Debe haber derecho a un medio ambiente sano y al desarrollo sustentable en donde se protejan los recursos naturales con una mejor regulación que evite su excesiva privatización.
			Providencia	Debe enfocar en la preservación de los recursos naturales y su impacto sobre las economías locales.

Table S15: Topic: Education

Determinant	Quartile	Topic Words	City	Text
Votes	Bottom 25	universal, acceso, público, oportunidad, laico, lucro, gratuito, integral, herramiento, educación pública, educación gratuita, calidad, educación.	Concón	Debe garantizar una educación gratuita y de calidad para el desarrollo integral de las personas sin exclusión.
			Vitacura	Debe ser obligatoria gratuita y de calidad distinguiendo lucro de abuso.
			Lo Barnechea	Debe ser gratuita de excelencia e inclusiva un derecho social desde la primera infancia para lograr una igualdad de oportunidades en el futuro.
	Top 25	superior, nivel, deber, educación cívica, deber haber educación, educación, gratis.	Canela	Debe haber educación pública gratuita y de calidad para todos y en todos los niveles de educación.
			Puyehue	Debe ser gratis y de buena calidad para todos/as especialmente los primeros 10 años.
			La Pintana	Debe haber acceso a la educación en todos sus niveles: básica media institutos profesionales universitaria laica democrática multicultural no quedando ningún chileno sin educación por falta de recursos.
Higher education	Bottom 25	deber, docente, educación, educación cívica, nivel, cívica, conocimiento, deber haber educación.	Combarbabá	Debe ser de libre acceso y de calidad en todos sus niveles y para todos.
			Placilla	Debe encargar el Estado que los ciudadanos estén informados respecto de como funciona la sociedad en que vivimos debiendo considerar la educación cívica durante toda la vida para que sean los ciudadanos participantes de lo que se realice en su país.
			Collipulli	Debe haber educación de calidad y gratuita para todas y todos.
	Top 25	educación gratuita, integral, lucro, superior, laico, educación pública, público, universal, educación, calidad, gratuito, acceso.	Valdivia	Debe entregar una educación sin lucro laica gratuita de calidad asegurando el acceso a la cultura.
			Valparaíso	Debe ser completa integral y mas profunda sin limitarse sólo a la educación formal.
			Antofagasta	Debe haber educación abierta e igualitaria de calidad con acceso garantizado a todos los chilenos y chilenas.
Pop. density	Bottom 25	deber haber educación, profesor, deber, profesional, gratuidad, educación cívica, cívico, educación gratuita.	Chile Chico	Debe garantizar el acceso a una educación gratuita y de calidad con carácter público.
			Tierra Amarilla	Debe haber educación gratuita y no salir endeudados..
			Pozo Almonte	Debe ser ser gratuita y de calidad con profesores con vocación y bien remunerados.
	Top 25	acceso, gratuito, laico, lucro, herramienta, educación pública, universal, integral, nivel, público, calidad, educación.	Viña del Mar	Debe garantizar acceso a la educación de calidad en todos sus niveles.
			Puente Alto	Debe haber acceso equitativo gratuito y de calidad a la educación fortaleciendo a la educación pública y sus profesores.
			Talcahuano	Debe ser gratuita universal permitiendo que cada persona se eduque hasta donde sus capacidades lo otorguen.
SEDI	Bottom 25	deber, sistema, docente, deber haber educación, educación cívico, educación digno, cívico, educación gratuito.	Monte Patria	Debe corregir abuso de alumnos frente al docente a los profesores se les han quitado atribuciones dentro del aula.
			Chanco	Es una buena educación de calidad para que las nuevas generaciones hagan de la nación un lugar mejor.
			San Nicolás	Debe existir una educación digna y de calidad para todos sin importar la calse social o ingresos económicos de las personas.
	Top 25	educación público, nivel, herramienta, integral, lucro, laico, educación, calidad, público, gratuito, universal, acceso.	Calama	Debe otorgar acceso a una educación pública gratuita laica y de calidad que alcance hasta la educación universitaria.
			Las Condes	Debe haber educación de calidad desde la enseñanza parvularia hasta la universidad o educación superior y que sea sin lucro para las entidades que administran las instituciones.
			Providencia	Debe ser gratuita y de calidad en todos sus niveles con el deber de ingreso a la educación formal.

Table S16: Topic: Security

Determinant	Quartile	Topic Words	City	Text
Votes	Bottom 25	familia, espacio, vivir, poder, comunidad, lugar, seguro, entorno, paz, familiar, violencia, tranquilo.	Concón	Debe cumplir requsitos básicos ya que es lugar donde se desarrolla la vida familiar que es el núcleo de nuestra sociedad.
			Vitacura	Debe consagrar la dignidad de las personas y el respeto asegurando en ellas el derecho a convivir en paz y tranquilidad.
			Lo Barnechea	Es agradable para todos los ciudadanos vivir en un estado seguro que permita a todos vivir tranquilos.
	Top 25	poder vivir, mujer, sentir, deber, hogar, casa, seguridad, mejorar.	Canela	Debemos vivir en paz conociendo y respetando los derechos y deberes; educando mejorando la comunicación en casa y a todo nivel cambiando los formatos de tv no más violencias; sino programas culturales educativos.
			Puyehue	Debe buscar seguridad de la familia como fin.
			La Pintana	Debe existir más vigilancia en las calles unión de vecinos y luminaria evitar violencia contra las mujeres y publicidad sexista.
Higher education	Bottom 25	mujer, mejorar, calle, tranquilo, deber, deber vivir, casa, poder vivir.	Combarbabá	Merecemos tener un lugar donde vivir y criar a nuestros hijos un hogar digno y seguro.
			Placilla	Debe dar seguridad inclusión e integración a la sociedad respetando la dignidad del ser humano.
			Collipulli	Debe ser derecho para todos el vivir tranquilamente y que nadie pase a llevar nuestra integridad física o psíquica.
	Top 25	sentir, seguro, lugar, violencia, paz, familiar, seguridad, comunidad, vivir, poder, familia, espacio.	Valdivia	Es vivir una vida sin violencia y que el estado garantice la seguridad de nuestros derechos.
			Valparaíso	Debe haber seguridad ciudadana dado que es importante para poder vivir y desarrollarnos en paz.
			Antofagasta	Debe sentir seguridad en el lugar en que uno este.
Pop. density	Bottom 25	violencia, deber vivir, seguridad, casa, vivir tranquilo, entorno, deber, miedo, tranquilo.	Chile Chico	Debe garantizar la seguridad entendiéndola como un derecho social.
			Tierra Amarilla	Debe penalizar a quienes agredan a personas física o psícamente pues nadie puede irrespetar la integridad física y psíquica de los ciudadanos.
			Pozo Almonte	Debe proteger y resguardar a la mujer ya que hoy esta en desigualdad en muchos ámbitos como la violencia física salario.
	Top 25	poder vivir, vivir, poder, seguro, paz, lugar, sentir, hogar, comunidad, familia, espacio.	Viña del Mar	Debe poder vivir tranquilos en nuestro barrio y casa sintiendonos protegidos por la justicia y derechos.
			Puente Alto	Debe poder disfrutar de cada espacio público de manera segura garantizando vivir en un país sin violencia.
			Talcahuano	Debe procurar a todos los habitantes un medio seguro y una vida pacifica que asegure el desarrollo optimo de la comunidad y la persona.
SEDI	Bottom 25	tranquilo, casa, poder vivir, poder, mejorar, calle, deber vivir, deber.	Monte Patria	Debe haber seguridad porque todos merecemos respeto y ya no podemos vivir tranquilos.
			Chanco	Debe contar con políticas de seguridad para evitar maltratos y robos.
			San Nicolás	Debe resguardar el derecho de seguridad permitiendo que las personas se desarrollen completamente aportando instancias en la sociedad.
	Top 25	miedo, sentir, paz, vivir, seguro, comunidad, familiar, violencia, lugar, seguridad, familia, espacio.	Calama	Necesitamos una vida sin violencia y tranquilidad disfrutando en todo ámbito con espacios de seguridad.
			Las Condes	Debe estar seguro en espacios públicos y privados.
			Providencia	Debe erradicar el miedo estando libre de violencia de autoridades con paz y tranquilidad.

Table S17: Topic: Equality

Determinant	Quartile	Topic Words	City	Text
Votes	Bottom 25	igualdad, ley, acceso, género, tratar, privilegio, proceso, deber, condición, diferencia, igual, oportunidad, deber haber igualdad.	Concón	Debe haber igualdad en el acceso las oportunidades meritocracia y no “pitutos” y ante la ley.
			Vitacura	Somos iguales ante la ley al acceso a la justicia y al debido proceso.
			Lo Barnechea	Es ser todos iguales ante la ley nadie puede ser tratado de manera diferente.
	Top 25	trato, justicia, clase social, clase, mujer, mismo, hombre.	Canela	Debe tener la posibilidad de defensa y trato de la misma forma ante la ley sin importar si tenemos o no plata.
			Puyehue	Hay brecha de sueldos entre hombres y mujeres además la competencia beneficia a profesores y técnicos y no a obreros habiendo nula posibilidad laboral de transexuales.
			La Pintana	Deben ser tratados igual sin importar la clase social.
Higher education	Bottom 25	mujer, hombre, igual, deber, clase, deber haber igualdad, género.	Combarbabá	No debe ser un obstaculo para el mundo laboral ya que el sexo femenino tiene las mismas capacidades laboras que el masculino.
			Placilla	Debe cumplir este derecho de igual manera ante la ley independiente de la clase socio-económica o cargo.
			Collipulli	Debe haber igualdad de género en la comunicación en el trato el respeto el trabajo y sueldo pues existe mayor valoración del hombre.
	Top 25	importar, tratar, existir, deber existir, privilegio, oportunidad, proceso, justicia, trato, mismo, diferencia, ley, igualdad.	Valdivia	No deben existir diferencias ni privilegios pues somos todos iguales.
			Valparaíso	Entiende como un todo incluyendo la igualdad ante la ley de acceso a la justicia y al debido proceso ante las cargas públicas y proporcionalidad ante los tributos.
			Antofagasta	Debemos ser juzgados de igual manera con justicia imparcial.
Pop. density	Bottom 25	importar, género, político, poder, deber, mismo derecho.	Chile Chico	Debe ser un salario sin distinción de género.
			Tierra Amarilla	Debe haber igualdad para sentirnos todos con los mismos derechos y oportunidades.
			Pozo Almonte	Debe impulsar fuentes de trabajo eliminando el fuero político.
	Top 25	privilegio, proceso, justicia, judicial, oportunidad, tratar, deber existir, acceso, ley, trato, existir, mismo, igualdad.	Viña del Mar	Debe haber igualdad ante género ante la ley acceso a la justicia y un debido proceso a desempeñar un cargo público etc siendo fundamentalmente al trato con igualdad y sin discriminación en su más amplio sentido.
			Puente Alto	Debe haber un mismo trato ante la ley poseyendo todos la misma condición.
			Talcahuano	Deben tener acceso a un proceso judicial que corresponda.
SEDI	Bottom 25	género, deber, discriminación, existir, deber existir, existir igualdad, mismo oportunidad, juzgar, mismo derecho.	Monte Patria	Debemos ser individuos con las mismas oportunidades.
			Chanco	Debe dar oportunidades por igual a todas las personas sin discriminacion ni privilegios.
			San Nicolás	Debe garantizar la igualdad de género siendo necesario terminar con los abusos ya que hoy podemos afirmar con gran determinación que no existen diferencias entre distintos géneros.
	Top 25	deber haber igualdad, proceso, mismo, trato, privilegio, tratar, justicia, acceso, oportunidad, ley, igualdad.	Calama	Debe contemplar las mismas normas o leyes para todas las personas sin que existan privilegios ni titulos nobiliarios.
			Las Condes	Debe haber igualdad ante la ley acceso a la justicia y el debido proceso igualdad frente a tributos y cargas públicas.
			Providencia	Debe tratar como igualdad de madios contemplando el concepto de equidad por el que no debe darse a todos lo mismo necesariamente.

Table S18: Sentiment analysis for two variables of topic “Security”.

word	value	sentiment
socioeconomic development		
tranquilo	98.68 %	Positive
casa	0.00 %	Neutral
poder vivir	96.71 %	Positive
poder	0.00 %	Neutral
mejorar	98.24 %	Positive
calle	0.00 %	Neutral
deber vivir	91.35 %	Positive
deber	0.00 %	Neutral
miedo	-94.96 %	Negative
sentir	96.94 %	Positive
paz	98.24 %	Positive
vivir	96.08 %	Positive
seguro	97.81 %	Positive
comunidad	0.00 %	Neutral
familiar	96.94 %	Positive
violencia	-98.14 %	Negative
lugar	0.00 %	Neutral
seguridad	97.81 %	Positive
familia	91.91 %	Positive
espacio	0.00 %	Neutral
Primary Economic Activity		
establecer	0.00 %	Neutral
seguridad social	98.26 %	Positive
deber establecer	0.00 %	Neutral
ciudadano	0.00 %	Neutral
hogar	0.00 %	Neutral
paz	98.24 %	Positive
seguridad	97.81 %	Positive
sentir	96.94 %	Positive
seguro	97.81 %	Positive
poder vivir	96.71 %	Positive
violencia	-98.14 %	Negative
deber haber grande	90.43 %	Positive
deber vivir	91.35 %	Positive
puerta	0.00 %	Neutral
delincuencia	0.00 %	Neutral
poder	0.00 %	Neutral
lugar	0.00 %	Neutral
vivir	96.08 %	Positive

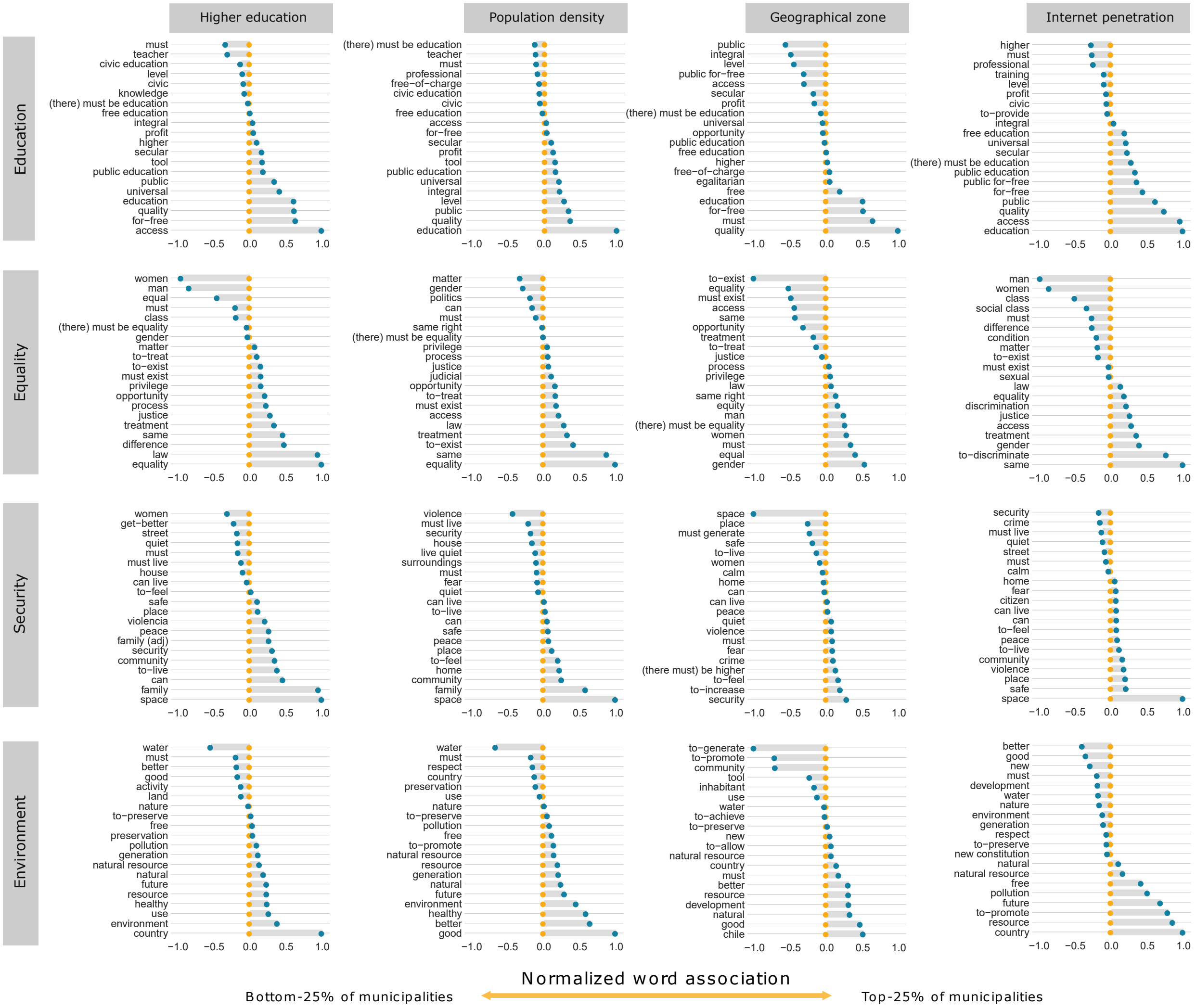

Figure S7: A word comparison of the constitutional rights debate, at the municipality level. We show the emergent topics: Education, Equality, Security, and Environment for four different citizen participation determinants. Words are oriented along the X-axis based on how much they are associated to the inspected determinant. We note that for the topic Equality, the word “process” comes from “due process” and “treatment” refers to “behaviour towards”. Likewise, for the topic Security, “door” comes from “revolving door”, which refers to inmate release and recidivism.

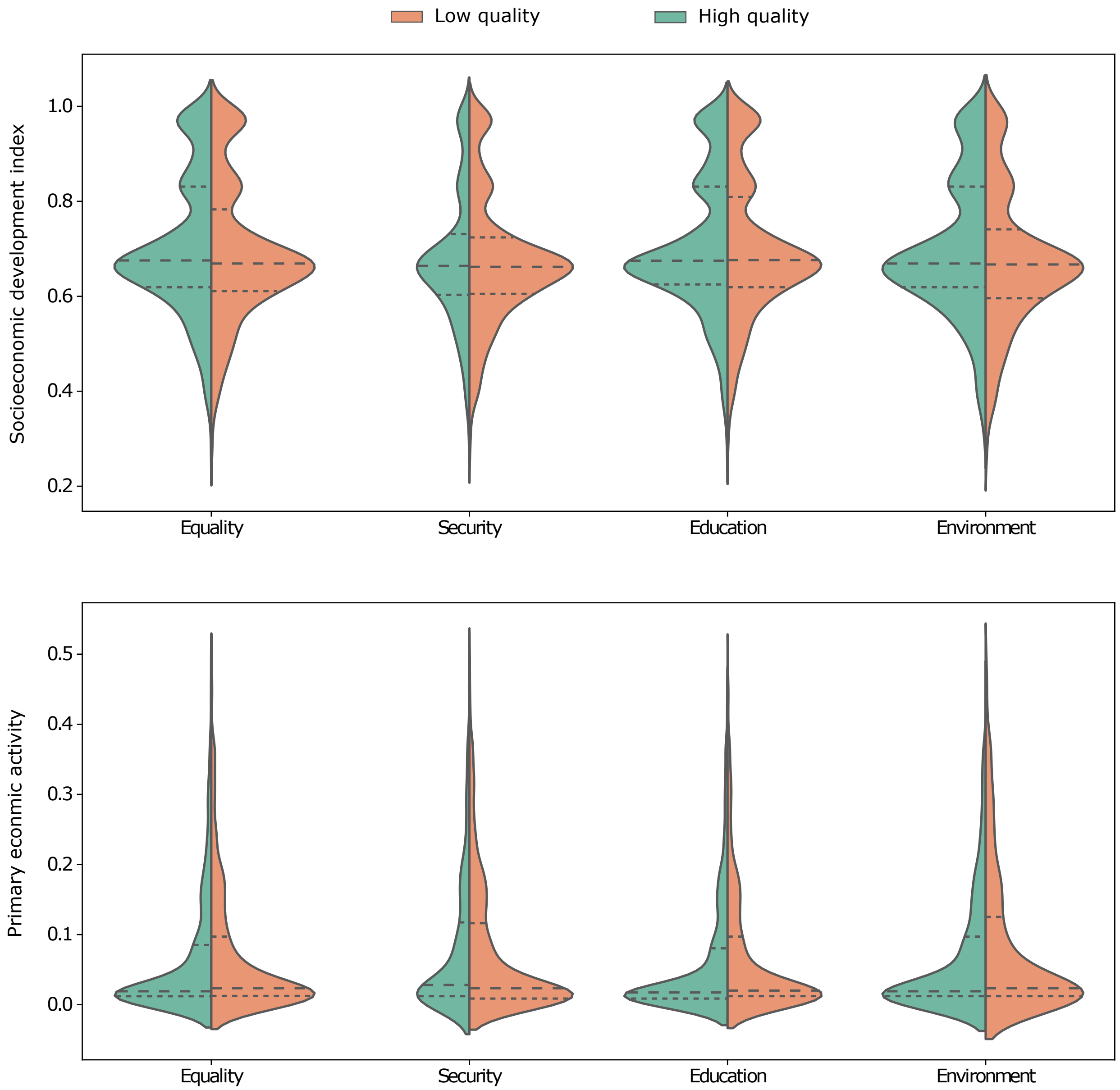

Figure S8: Distribution of quality argument for topics Equality, Security, Education and Environment, and two variables: socioeconomic development index (top panel) and share of primary economic activity (bottom panel). Dashed lines represent quartiles.

References

1. Wallach HM, Murray I, Salakhutdinov R, Mimno D. Evaluation methods for topic models. In: Proceedings of the 26th annual international conference on machine learning; 2009. p. 1105–1112.
2. Taddy M. On estimation and selection for topic models. In: Artificial Intelligence and Statistics; 2012. p. 1184–1193.
3. Mimno D, Wallach HM, Talley E, Leenders M, McCallum A. Optimizing semantic coherence in topic models. In: Proceedings of the conference on empirical methods in natural language processing. Association for Computational Linguistics; 2011. p. 262–272.
